# Transport mechanism of the SLC4 proteins—Lessons from recent structural and computational studies

**DOI:** 10.1016/j.jbc.2026.113092

**Published:** 2026-05-15

**Authors:** Hristina R. Zhekova, Alexander Pushkin, Weiguang Wang, Liyo Kao, Kirill Tsirulnikov, Rustam Azimov, Natalia Abuladze, Dora Acuna, Z. Hong Zhou, D. Peter Tieleman, Ira Kurtz

**Affiliations:** 1 Centre for Molecular Simulation, Department of Biological Sciences, University of Calgary, Calgary, Canada; 2 Division of Nephrology, David Geffen School of Medicine, Department of Medicine, University of California, Los Angeles, California, USA; 3 Electron Imaging Center for Nanosystems, California NanoSystems Institute, University of California, Los Angeles, California, USA; 4 Department of Microbiology, Immunology and Molecular Genetics, University of California, Los Angeles, California, USA; 5 Brain Research Institute, University of California, Los Angeles, California, USA

**Keywords:** bicarbonate transport, cryogenic electron microscopy, lipid–protein interaction, membrane protein, molecular modeling, protein conformation, secondary transporter, SLC4 family, structure-function, X-ray crystallography

## Abstract

The solute carrier family 4 (SLC4) secondary transporters are found ubiquitously in animal, plant, and fungal tissues, where they transport HCO_3_^−^, CO_3_^2^^−^, borate, and other ions, necessary for pH maintenance and ion homeostasis. Dysfunction in SLC4 members has been related to debilitating human diseases and impaired crop growth. SLC4 proteins feature high transport rates and multiple transport modes (cation–anion symport and cation-dependent and -independent anion exchange) achieved through small sequence differences in key structural regions of the protein. This has made them a subject of increasing scientific interest. In the last decade, X-ray and cryogenic electron microscopy structures of SLC4 members have become available, prompting computational modeling of their function. Structures of selected SLC4 members in outward facing, occluded/intermediate, and inward facing conformations, together with computational modelling support an elevator transport mechanism with small vertical translocation and protein reorganization. However, inward-facing structures of the Na⁺-dependent SLC4 transporters and true intermediate experimental structures for all mammalian SLC4 members have not yet been determined. Binding sites for ions, lipids, and inhibitors were identified in several family members, providing insights into regulation and the differences between the cation-dependent and -independent transport. SLC4 complexes with intracellular proteins revealed how protein–protein interactions impact the structure of anion exchanger 1 and the relative position of its cytoplasmic and transmembrane domains. This review summarizes these structural and computational insights, places them in the context of the SLC4 transport mechanism, and highlights questions for further structural, functional, and computational studies.

The mammalian solute carrier family 4 (SLC4) consists of 10 secondary transporters involved in the transport of HCO_3_^−^, CO_3_^2^^−^, Cl^−^, Na^+^, K^+^, and H^+^ that play key roles in critical physiological processes, including cell and extracellular pH regulation, acid–base equivalent reabsorption and secretion, ion transport, and cell volume regulation. These transporters are widely expressed in erythrocytes, brain, eye, heart, kidney, lung, pancreas, small intestine, large intestine, and testis (1, 2, 3, 4).

Phylogenetically, the conserved 7 + 7 inverted-repeat transmembrane fold of the SLC4 transporters is shared by animal carbonate/bicarbonate transporters and plant/fungal borate transporters, consistent with an ancient core architecture adapted to distinct substrates and regulatory modules. Functionally, a central role of SLC4 proteins is pH maintenance in the tissues in which they are expressed, where they interface with metabolism and signaling. They shape intracellular pH microdomains that tune enzyme activity and excitability. They also support systemic CO_2_ exchange [*via* anion exchanger 1 (AE1) in erythrocytes] and contribute to nutrient/toxin tolerance *via* boron export in plants and fungi (2, 5).

Mammalian SLC4 transporters have a variety of transport properties including their electrogenicity, and whether they function as uniporters, symporters, or exchangers with associated channel-like activity. These transporters also differ with regard to their ion transport specificity (2, 4, 6). The Na^+^-independent anion exchangers of the family AE1, AE2, and AE3 (encoded by *SLC4A1*, *SLC4A2*, and *SLC4A3*, respectively) exchange Cl^−^ for HCO_3_^−^ (7). AE1 is also historically known as Band 3 protein reflecting its abundance as the third major band on SDS–PAGE of red cell membranes (3). NBCe1 and NBCe2 (encoded by *SLC4A4* and *SLC4A5*, respectively) are electrogenic symporters mediating the transport of Na^+^-CO_3_^2^^−^. NBCn1 and NBCn2 (encoded by *SLC4A7* and *SLC4A10*, respectively) are electroneutral Na^+^-HCO_3_^−^ symporters (a 2Na^+^-CO_3_^2^^−^ transport mode is also possible) (1, 2). NBCn1 transport is also associated with a Na^+^ cation current, and NBCn2 is associated with a futile Cl^−^/Cl^−^ exchange process as part of its transport cycle (4, 8). NDCBE, the Na^+^-dependent chloride/carbonate exchanger encoded by *SLC4A8*, is an exchanger-like AE1-AE3; however, its transport is Na^+^ dependent where it functions in a Na^+^-CO_3_^2^^−^/Cl^−^ exchange mode (2, 9, 10). The function of AE4 (encoded by *SLC4A9*) remains controversial. The transporter was originally reported to be a Na^+^-independent Cl^−^/HCO_3_^−^ exchanger and subsequently a Na^+^-HCO_3_^−^ symporter. More recent evidence suggests that AE4 functions as a monovalent cation-dependent Cl^−^/HCO_3_^−^ exchanger (or potentially a Na^+^-CO_3_^2^^−^/Cl^−^ exchanger (2, 11)). Finally, BTR1 (encoded by *SLC4A11*), which has the lowest sequence identity with the remaining mammalian members of the family, initially was reported to be a borate transporter, but more recent studies have provided evidence for H^+^ transport, NH_3_-H^+^ symport, or allosteric NH_3_/NH_4_ activation of H^+^/OH^−^ transport (12, 13).

Multiple clinical disorders associated with abnormal function, expression, or membrane targeting of SLC4 transporters in humans are currently known (1, 2, 6, 10, 14, 15). Among these disease states are red blood cell morphological abnormalities, hemolytic anemia, and distal renal tubular acidosis (*via* SLC4A1, (3)); primary biliary cirrhosis, colon, liver, and gastric cancers, and osteopetrosis (*via* SLC4A2, (16, 17, 18)); seizures, epilepsy, cardiomyocyte hypertrophy, and short QT syndrome (*via* SLC4A3, (19, 20, 21, 22, 23, 24, 25)); proximal renal tubular acidosis and neurological and dental abnormalities (*via* SLC4A4, (1, 26, 27, 28)); hypertension and salt sensitivity, brain morphological abnormalities and reduced intracerebral volume, severe retinopathy, and inflammation associated with rheumatoid arthritis (*via* SLC4A5, (29, 30, 31, 32, 33)); cardiovascular disease, breast cancer, abnormal brain development, and synaptic excitability (*via* SLC4A7, (34, 35, 36, 37, 38, 39)); reduced neuronal excitability and delayed seizure onset (*via* SLC4A8, (2, 6, 40)); reduced salivary gland secretion and impaired renal sensing and pendrin-driven acid/base regulation (*via* SLC4A9, (11, 41)); autism and seizures (*via* SLC4A10, (15, 42)); and congenital hereditary endothelial dystrophy, Fuchs endothelial corneal dystrophy, and Harboyan syndrome (*via* SLC4A11, (43, 44)).

Other than the mammalian SLC4 transporters described above, SLC4 transporters involved in boron metabolism in plants and yeast have also received attention due to the strong detrimental impact of boron deficiency or excess on crop growth. These transporters likely export borate as [B(OH)_4_]^−^ in exchange for H^+^ (45, 46, 47, 48, 49). Examples of such transporters are the borate 1 transporters from *Arabidopsis thaliana, Oryza sativa,* and *Saccharomyces mikatae* (AtBor1 (45, 47, 48), OsBor1 (47), and Bor1p (49), respectively).

Since the identification of the first member of the family, AE1, more than 40 years ago, numerous studies have shed light on the structure-function relationship in the SLC4 family (3). Early structural studies provided limited information about the structure of the first few transmembrane helices (TMs), the cytoplasmic domain (CTD), and the N_t_ tail of Band 3 (50, 51, 52, 53, 54, 55, 56, 57, 58, 59). A major impetus for the field was the resolution of the outward facing (OF) structure of the transmembrane domain (TMD) of AE1 by Arakawa *et al.* in 2015 (60), which allowed for the accumulated functional data to be put in an unambiguous structural perspective (3). Advances in cryogenic electron microscopy (cryoEM) technologies and computational modeling opened additional successful avenues for exploration of the structure and function of the SLC4 proteins and resulted in recently published structures of the OF state of NBCe1 (61), NBCn1 (62), NDCBE (63), AE1 (64, 65, 66, 67, 68), AE2 (17, 18), AE3 (69), and BTR1 (43, 70), the inward facing state (IF) of AE1 (67, 68, 71), AE2 (17, 18), and BTR1 (43, 70), IF and occluded states of AtBor1 and Bor1p (45, 47, 48, 49), and identification of elevator-type transport in the family, during which a substrate-binding core domain moves as a largely rigid body relative to a more stationary gate/scaffold domain, translocating the binding site vertically across the membrane and alternately exposing it to either side of the membrane (17, 43, 45, 48, 67, 68, 71). Substrate, lipid, and inhibitor binding sites within the protein structures were resolved for several members of the family (17, 18, 43, 62, 63, 64, 65, 67, 68, 70). Computational modeling elucidated further the substrate and lipid binding and ion and water permeation trends within some of the X-ray and cryoEM structures and SLC4 homology models (62, 63, 68, 71, 72, 73, 74, 75, 76, 77). This review aims to summarize the most important findings from the recent structural and computational studies, to provide an up-to-date description of the structure-function relations in the SLC4 family, and to highlight areas in the SLC4 transport mechanism that require further structural, functional, and computational studies.

## Overall SLC4 structure

**Figure 1:**
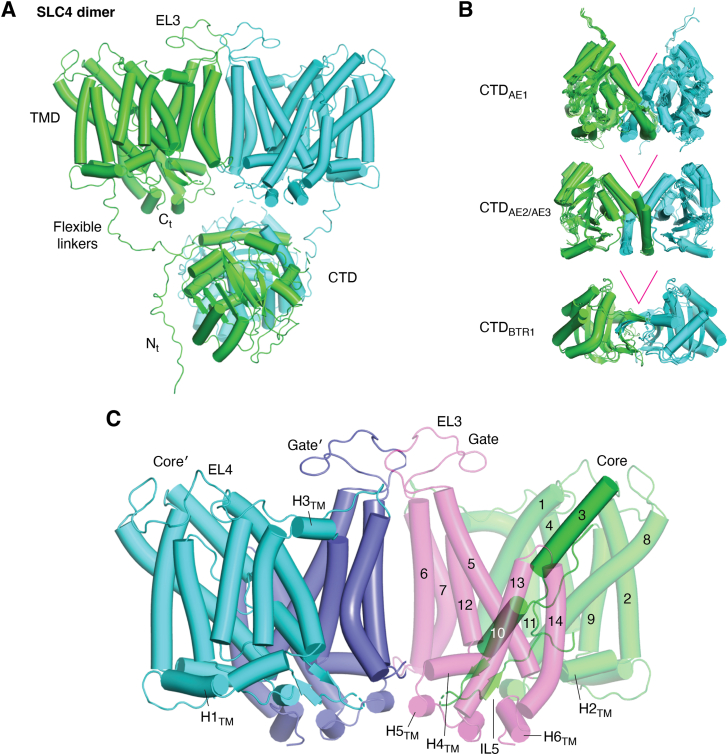
**SLC4 structure.***A*, overall protein structure of the SLC4 dimer (specifically, AE1 dimer from the PDB ID 7TVZ entry) highlighting the different parts of the SLC4 proteins discussed in the text—N_t_, CTD, flexible linker, TMD, and C_t_. The two monomers in the dimer are shown as *green* and *cyancylinders*. *B*, overlap of all CTD domains resolved for AE1 (*top*), AE2 and AE3 (*middle*), and BTR1 (*bottom*). The CTD monomers are shown as *cyan* and *green cylinders*. The CTD dimers are oriented in the V conformation and the V-shaped cleft formed at the dimeric interface is indicated. *C*, gate and core domains in the TMD with numbered TM helices. The rigid gate domain is shown as *purple* and *pink cylinders* (gate and gate' domains of monomers 1 and 2, respectively), while the core domains are shown as *green* and *cyan cylinders* (core and core' domains of monomers 1 and 2, respectively). AE1,2 and 3: anion exchangers 1, 2, and 3; BTR1, a human SLC4 transporter, corresponding to SLC4A11; CTD, cytoplasmic domain; SLC4, solute carrier family 4; TM, transmembrane helix; TMD, transmembrane domain; EL, extracellular loop; IL, intracellular loop; Ct, C-terminus; Nt, N-terminus; H1-6_TM_, 6 short helical sections from the EL and IL in the TMD.

**Figure 2:**
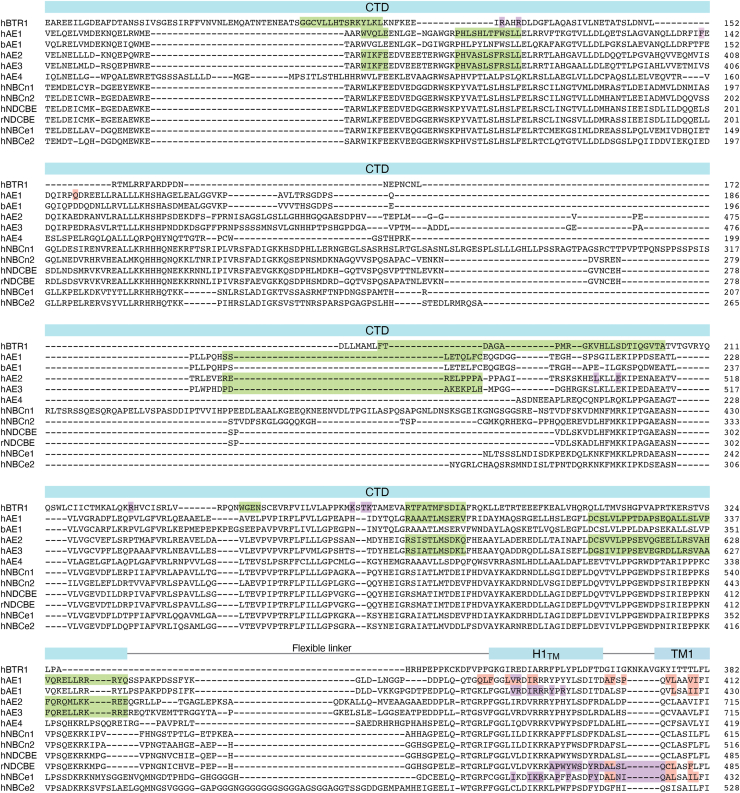

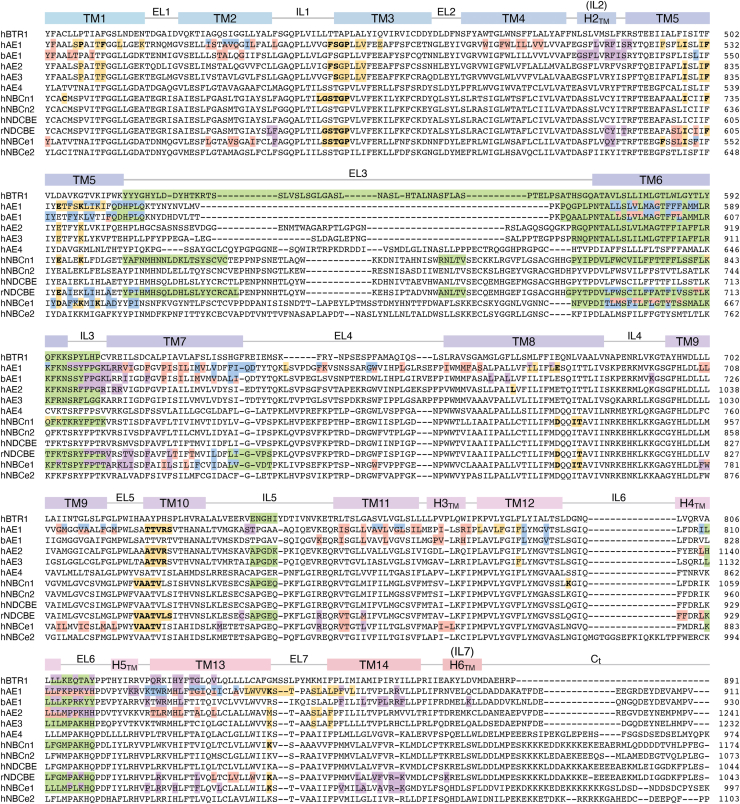
**Sequence alignment of the mammalian SLC4 proteins mentioned in the review.** The alignment includes the sequences of the 10 members of the human SLC4 protein family, together with bovine AE1 (bAE1) and rat NDCBE (rNDCBE) reported in the “Overall SLC4 Structure” section and Table 1. The entire N_t_ tail and the end of the C_t_ tail are excluded from the alignment for clarity. The position of the CTD, flexible linker, TMs, and loops forming the TMD, and the beginning of C_t_, corresponding to the human AE1 structure in PDB ID 7TVZ are also indicated. Due to the absence of CTD structures for the Na^+^-dependent transporters and the considerable difference between the CTD of AE1-AE3 *versus* CTD of BTR1, the alphahelical/beta sheet organization of the CTD is not indicated. Color coding: *green highlight*—dimerization domains (from cryoEM and X-ray structures), *orange highlight*—substrate and inhibitor binding sites [from cryoEM structures (*bold font*) and MD simulations], *red highlight*—cholesterol/cholesterol derivatives binding sites (from cryoEM structures and CGMD simulations), *blue highlight*—phospholipid binding sites (from cryoEM structures and CGMD simulations), and *purple highlight*—PIP_2_/PIP_2_ derivatives binding sites (from cryoEM structures, CGMD, and all-atom MD simulations). The following UniProt sequences were used for the alignment: Q8NBS3-1 for human BTR1, P02730 for human AE1, P04920 for human AE2, P48751 for human AE3, Q96Q91 for human AE4, Q9Y6M7 for human NBCn1, Q6U841 for human NBCn2, Q2Y0W8 for human NDCBE, Q6RVG2 for rat NDCBE, Q9Y6R1-2 for human NBCe1, and Q9BY07 for human NBCe2. The sequence corresponding to entry 8E34 from the PDB database was used for bovine AE1. The plant and yeast Bor1 proteins are not included in the alignment due to the lack of data on substrate and lipid binding residues obtained from structural or computational studies. Substrate binding sites of hAE4 determined from MD simulations have not been included in *Fig.2* due to lack of corroboration from cryoEM structural data. AE1,2,3,4: anion exchangers 1, 2, 3 and 4; BTR1, a human SLC4 transporter, corresponding to SLC4A11; CGMD, coarse-grained MD; cryoEM, cryogenic electron microscopy; CTD, cytoplasmic domain; MD, molecular dynamics; PIP_2_, phosphatidylinositol bisphosphate; SLC4, solute carrier family 4; TM, transmembrane helix; TMD, transmembrane domain; NBCn1, electroneutral Na^+^-carbonate symporter 1; NBCn2, electroneutral Na^+^-carbonate symporter 2; NDCBE, Na^+^-dependent chloride/carbonate exchanger, corresponding to SLC4A8; NBCe1, electrogenic Na^+^-carbonate symporter 1; NBCe2, electrogenic Na^+^-carbonate symporter 2; Bor1, borate transporter 1; IL, intracellular loop; EL, extracellular loop; H1-6_TM_, six short helical sections from the EL and IL in the TMD; MD, molecular dynamics.

The mammalian SLC4 structure can be divided into several regions—the N- and C-terminus tails (N_t_ and C_t_, respectively), the cytoplasmic domain CTD (also known as N-terminal cytoplasmic domain or NTD in some works), the transmembrane domain TMD, and the flexible linker between the CTD and the TMD (3). The plant and yeast SLC4 transporters Bor1 and Bor1p have the same TMD organization as the one found in the mammalian SLC4; however, they lack the CTD of the mammalian SLC4 members. Instead, the plant Bor1 transporters have a small CTD-like structure positioned between TMs 10 and 11 of the TMD (45, 47, 48). The different regions of the OF AE1 [PDB ID: 7TVZ (66), which is the most complete mammalian SLC4 structure resolved to date and includes part of N_t_, most of the CTD, the flexible linkers, the TMD, and part of C_t_] are displayed in Figure 1*A*. Sequence alignment of the 10 mammalian SLC4 family members, including either the principal isoforms in the UniProt database, or the isoforms of the reported human and non-human cryoEM structures is presented in Figure 2. The list of the available X-ray and cryoEM SLC4 structures reviewed in the present work is provided in Table 1.

**Table 1:** Atomistic SLC4 structures resolved by X-ray diffraction, solution NMR, or cryoEM used in the review

Protein (Ref.)	Resolution	Conf. state	Part of protein	PDB ID
Human AE1 (53)	NMR	N/A	TM1, TM2	1BTQ, 1BTR, 1BTS, 1BTT
Human AE1 (54)	NMR	N/A	Nt (first 15 residues)	2BTA, 2BTB
Human AE1 (55)	NMR	N/A	Nt (first 15 residues)	3BTB
Human AE1 (56)	NMR	N/A	Loop of TMs 12/13	1BH7
Human AE1 (57)	NMR	N/A	TM1	1BNX
Human AE1 (58)	X-ray, 2.6 Å	N/A	CTD	1HYN
Human AE1 (59)	X-ray, 2.1 Å	N/A	CTD	4KY9
Human AE1 (60)	X-ray, 3.5 Å	OF-OF	TMD	4YZF
Bovine AE1 (71)	cryoEM, 4.4 Å	IF-IF	TMD	8D9N
	cryoEM 6.3 Å	IF-IF	TMD + CTD	8EEQ
	cryoEM 6.0 Å	IF-OF	TMD + CTD	8E34
Human AE1 (65)	cryoEM, 2.35 Å	OF-OF	TMD	7UZ3
	cryoEM, 2.40 Å	OF-OF	TMD + CTD + Nt	7V0K
	cryoEM, 2.50 Å	N/A	CTD	7UZV
	cryoEM, 2.80 A	OF-OF	TMD	7V07
	cryoEM, 2.74 A	OF-OF all	TMD + CTD + Nt	8CS9
	cryoEM 2.70–3.00 Å	N/A	CTD	7V0T, 7V0U, 7VOY
	cryoEM, 3.00–3.30 Å	OF-OF all	TMD	8CRQ, 7V19, 8CRR
	cryoEM, 3.00 Å	OF-OF	TMD	8CRT
	cryoEM, 2.90 Å	OF-OF	TMD + CTD + Nt	8CTE
	cryoEM, 2.70 Å	N/A	CTD	8CSY
	cryoEM, 3.30 Å	OF-OF	TMD	8CT3
Human AE1 (66)	cryoEM, 4.80 Å	OF-OF	TMD + CTD	7TW2
	cryoEM, 4.60 Å	OF-OF	TMD + CTD	7TW0
	cryoEM, 4.60 Å	OF-OF	TMD + CTD	7TW1
	cryoEM, 3.60 Å	OF-OF	TMD + CTD	7TVZ
	cryoEM, 4.40 Å	OF-OF	TMD + CTD	7TW3
	cryoEM, 5.70 Å	OF-OF	TMD + CTD	7TW5
	cryoEM, 5.70 Å	OF-OF	TMD + CTD	7TW6
Human AE1 (64)	cryoEM, 2.99 Å	OF-OF	TMD	7TY4
	cryoEM, 3.37 Å	OF-OF	TMD	7TY7
	cryoEM, 2.95 Å	OF-OF	TMD	8T6V
	cryoEM, 2.98 Å	OF-OF	TMD	7TY6
	cryoEM, 3.07 Å	OF-OF	TMD	7TYA
	cryoEM, 3.13 Å	OF-OF	TMD	8T6U
	cryoEM, 3.18 Å	OF-OF	TMD	7TY8
Human AE1 (67)	cryoEM, 2.99 Å	IF-IF	TMD	8T3U
	cryoEM, 2.97 Å	IF-OF	TMD	8T3R
	cryoEM, 3.12 Å	IF-OF	TMD	8T44
	cryoEM, 2.99 Å	IF-IF	TMD	8T45
	cryoEM, 3.16 Å	OF-OF	TMD	8T47
Human AE1 (68)	cryoEM, 2.40 Å	OF-IF	TMD	9MND
	cryoEM, 3.11 Å	OF-OF	TMD	9MNG
	cryoEM, 2.88 Å	IF-IF	TMD	9MOS
Human AE2 (17)	cryoEM, 3.32 Å	IF-IF	TMD + Ct + CTD	8GVH
	cryoEM, 3.08 Å	OF-OF	TMD	8GV8
	cryoEM, 3.06 Å	IF-IF	TMD + CTD	8GV9
	cryoEM, 3.25 Å	IF-IF	TMD	8GVA
	cryoEM, 2.89 Å	OF-OF	TMD	8GVC
	cryoEM, 3.17 Å	IF-OF	TMD	8GVE
Human AE2 (18)	cryoEM, 3.20 Å	IF-IF	TMD	8JNI
	cryoEM, 3.30 Å	IF-IF	TMD + CTD	8JNJ
Human AE3 (69)	cryoEM, 2.73 Å	OF-OF	TMD	8Y85
	cryoEM, 2.75 Å	OF-OF	TMD	8Y86
	cryoEM, 2.89 Å	OF-OF	TMD	8Y8K
	cryoEM, 3.35 Å	IF-IF	AE2 TMD + AE3 CTD	8ZLE
Human NBCe1 (61)	cryoEM, 3.90 Å	OF-OF	TMD	6CAA
Rat NDCBE (63)	cryoEM, 3.40 Å	OF-OF	TMD	7RTM
Human NBCn1 (62)	cryoEM, 3.30 Å	OF-OF	TMD	9OVR
Human BTR1 (43)	cryoEM, 2.94 Å	OF-OF	TMD + CTD	7X1I
	cryoEM, 2.94 Å	IF-IF	TMD + CTD	7X1G
	cryoEM, 2.96 Å	IF-IF	TMD + CTD	7X1H
	cryoEM, 2.84 Å	OF-OF	TMD + CTD	7X1J
Human BTR1 (70)	cryoEM, N/A	IF-IF	TMD + CTD	21KY
	cryoEM, N/A	OF-OF	TMD + CTD	21KZ
AtBor1 (45)	X-ray, 4.11 Å	Occ.	TMD	5L25
AtBor1 (48)	cryoEM, 2.15 Å	IF	TMD	8TEG
	cryoEM, 2.30–3.02 Å	IF-IF all	TMD	8TEM, 8TEH, 8TEI
	cryoEM, 2.55 Å	Occ.	TMD	8TEJ
	cryoEM, 2.77 Å	Occ. – Occ.	TMD	8TEL
	cryoEM, 2.98 Å	IF – Occ.	TMD	8TEN
Bor1p (49)	cryoEM, 5.90 Å	IF-IF	TMD	5SV9

The BTR1 structures from reference (70) were still held for publication at the time that this review was revised, and their resolution is not included in Table 1.

The N_t_ and C_t_ termini are among the regions with the lowest sequence identity in the SLC4 family, which likely reflects member-specific interactions of these regions with the intracellular environment. The N_t_ tail of AE1 includes a phosphorylation site at Y8 involved in phosphorylation-dependent interactions with cytoplasmic proteins such as aldolase or glyceraldehyde 3-phosphate dehydrogenase (54, 55). Solution nuclear magnetic resonance structures of the first 15 residues of the AE1 N_t_ (54, 55) and cryoEM structures of the 2 to 31 N_t_ residues of AE1, where one of the N_t_ tails of an AE1 dimer is resolved in complex with ankyrin (65), demonstrate that the N_t_ tail adopts different conformations, depending on its local environment. The C_t_ tail of AE1 is involved in an interaction with carbonic anhydrase II, necessary for the CO_2_/HCO_3_^−^ metabolism in the body (78, 79), although no structures of the AE1-carbonic anhydrase II complex have been resolved yet. The C_t_ tail has been resolved in an AE2 structure, where it is positioned in the IF cavity of the TMD and the terminal carboxylate group is bound at the central binding site in a configuration that resembles a self-inhibited state (17) as well as in a self-inhibited state of AtBor1 in which the C_t_ tail of the neighboring monomer in the dimer obstructs entry to the IF cavity (48). In NBCe1-B, PIP_2_ (phosphatidylinositol bisphosphate)-binding regulatory site was found in the area of positively charged residues 37 to 65 from the N_t_ tail, which is part of the autoinhibitory domain of NBCe1-B. Inositol 1,4,5-trisphosphate binding protein interacts with the N_t_ of NBCe1-B reversing its autoinhibition effect (80).

The CTD in the mammalian SLC4 proteins is a dimer made of two globular subunits, each of which usually consist of ∼300 amino acid residues (Fig. 2) although variation in the CTD size is common even within the isoforms of the same SLC4 proteins. For instance, NBCn1 (the SLC4A7 member of the family) features more than 12 isoforms, some of which lack the cassette 2 region of 124 residues from the N_t_ and CTD, leading to considerable differences in overall protein length and CTD size (81). Currently, CTD structures have been resolved for AE1 (58, 59, 65, 66), AE2 (17, 18), AE3 (69), and BTR1 (43, 70) with multiple missing sections, due to high structural flexibility in the regions of lower sequence identity. The CTD of the anion exchangers and BTR1 are shown in Figure 1*B*. They are composed of several short α-helical sections and β-sheet strands; however, the number and orientation of these sections and strands may be different in the different SLC4 members. The available CTD structures of AE2 and AE3 show high structural similarity [root mean square deviation RMSD_CTD_(AE2 *versus* AE3) of 0.48–1.11 Å] and their monomers follow generally the structure of the AE1 CTD (Figs. 1*B* and 2). The CTD of BTR1 has a unique sequence among the SLC4 proteins and is significantly smaller (about 270 residues compared to the 290–315 residues in AE1, AE2, and AE3), Figure 2. It features a considerably different dimerization interface from the one found in the AE1-3 CTD structures (Fig. 1*B*, also see below).

The flexible linkers are long unstructured regions of about 30 to 40 residues, although in some members of the family like NBCe2, this region can reach 60 residues (Fig. 2). The flexible linkers have been resolved only in a single AE1 structure where CTD motion was arrested *via* interactions with the intracellular protein 4.2 (PDB ID: 7TVZ) (66).

The TMD, which is the region with the highest sequence identity among the different SLC4 members (Fig. 2), has the same overall organization in all resolved SLC4 TMD structures. It features a 7 + 7 inverted repeat transmembrane helical fold that can be divided further into a gate (rigid dimerization scaffold) and core (transport) domain (Fig. 1, *B* and *C*) (3). The gate domain consists of TMs 5 to 7 and 12 to 14, while the core domain includes TMs 1 to 4 and 8 to 11. The gate and core domains are connected *via* two short helical sections [H2_TM_ and H3_TM_ in AE1 (3)], which lie in the plane of the membrane bilayer at the membrane interface with the intra- and extra-cellular space, respectively. Another, longer, helix (H1_TM_) is positioned at the membrane/cytoplasm interface, parallel to the membrane plane, and anchors the flexible linker between the CTD and TMD to the TMD. Two short helices, TMs 3 and 10, span the lipid membrane half-way and their N termini meet at the center of the protein to form an area of great functional importance, which includes the central substrate binding sites (3, 17, 43, 62, 63, 64, 68, 72). The TMD features 7 extracellular loops (ELs) and 7 intracellular loops (ILs). The extracellular loop 3 (EL3) varies substantially in size between the Na^+^-independent anion exchangers, on the one hand, and the Na^+^-dependent transporters of the family and AE4 on the other hand (Fig. 2). It was fully resolved in recently reported NDCBE and NBCn1 cryoEM structures (62, 63) and is composed of four short helical portions and a β-sheet section stabilized with two Cys-Cys bonds (C636-C684 and C638-C672 in NDCBE and C766-C814 and C768-C802 in NBCn1) with two N-glycosylation sites at N646 and N666 in NDCBE and three N-glycosylation sites at N776, N786, and N796 in NBCn1. The EL3 loop and its purported glycosylation sites have not been resolved in the reported AE2, AE3, and BTR1 structures. A well-known N-glycosylation site, however, was resolved in multiple cryoEM structures of AE1, at N642 of EL4 (64, 65, 66). The intracellular loop 5 (IL5) is another loop of interest. In the OF states, it manifests as a β-hairpin, as observed after structural relaxation with molecular dynamics (MD) (71, 72). The entire β-hairpin is flexible and involved in interactions with the TMs of the same monomer, the adjacent monomer, lipids in the region, and even the CTD, where IL5 purportedly acts as an anchor to stabilize the CTD in space, as seen in several IF AE2 structures (17, 18). Several cryoEM structures of IF AE1 have been resolved with shorter IL5, unfolded TM10, and elongated TM11, which has accommodated some of the IL5 residues (67, 68, 71). In the plant borate transporters, a small CTD-like structure of about 100 residues is found between TM10 and TM11. This CTD was resolved in recent occluded AtBor1 states and demonstrates a long helix composed of the entire TM10 and the beginning of the CTD, while the IF Bor1 states were resolved with a standard TM10 and a beginning of the CTD that is similar to the β-hairpin of IL5 in the mammalian SLC4 structures (48). Thus, the mammalian cytoplasmic loop (IL5) or plant CTD-like structure between TMs 10 and 11, together with TMs 10 and 11, have been implicated as an important part of the OF ↔ IF conformational process in both mammalian and plant SLC4 (48, 71).

## SLC4 proteins are dimers

Oligomerization is frequently observed in secondary transporters and has been discussed as arising from the need of structural stability and functional and allosteric regulation without excessive genome expansion (61, 82, 83). Although no structural or computational studies have tackled the specific role of oligomerization in the SLC4 transport cycle, earlier studies have provided evidence that AE1 assembles in different oligomeric forms, depending on the detergents used during the solubilization process (50). The most prevalent SLC4 oligomers were dimers; however, higher-order oligomers, such as trimers, tetramers, and hexamers have also been observed in detergents such as polyoxyethylene dodecyloethers, pentaoxyethylene alkylethers, octaethylene glycol monododecyl ether, and n-octylpolyoxyethylene, depending on the temperature and oligomerization time. In red blood cells, AE1 has been found to bind to ankyrin as a tetramer or a pseudotetramer, with ankyrin binding two AE1 dimers in several studies (84). In the only *in vivo* study of SLC4 proteins, it was demonstrated using spatial intensity distribution analysis in the native rat kidney that the predominant population of NBCe1-A is dimeric (85).

The first CTD and TMD structures of AE1 were resolved as dimers providing direct structural evidence for this preferred oligomeric state (58, 59, 60). More recent structures of native complexes isolated from human red blood cell (RBC) membranes reveal that AE1 exists primarily as dimers, which serve as foundational platforms for the ankyrin complex. These dimers recruit protein 4.2 and ankyrin, enabling the formation of higher-order supracomplexes. Ankyrin bridges two AE1 dimers *via* distinct repeat domains (ARs 6–13 to protein 4.2; ARs 17–20 to a second AE1 dimer) (66). This suggests a stepwise assembly model during erythropoiesis, with AE1 dimers acting as core scaffolds that nucleate cytoskeletal attachment and ensure mechanical stability of the RBC membrane. Intact ankyrin-1 complex purified from RBC ghosts revealed a core assembly with one central AE1 dimer (Band 3-I) tightly bound to protein 4.2 and ankyrin, flanked by two additional AE1 dimers (Band 3-II and -III) in larger classes (65). Subtomogram averaging of native membrane vesicles confirmed this organization *in situ*, showing up to three AE1 dimers. The T-shaped ankyrin conformation facilitates lateral clustering of membrane proteins, including RhAG/RhCE and aquaporin-1, underscoring the dual role of AE1 in ion homeostasis and structural scaffolding within the native RBC membrane.

To date, more than 60 structures of SLC4 proteins, including AE1, AE2, AE3, NBCe1, NBCn1, NDCBE, BTR1, AtBor1, and Bor1p have been resolved (Table 1), all in the dimeric state, with both the TMD and CTD (where available) involved in the dimerization interactions (Fig. 2). The TMD dimerization interface is consistent between the mammalian and plant/fungal SLC4 family members and mostly involves the hydrophobic portions of TM6 [Fig. 2, Ref. (48)]. Parts of the EL3, ILs 3, 5, and 6, and H4_TM_ also participate in the dimeric stabilization, with prominent hydrophobic interactions between IL3 and H4_TM_. CTD dimeric structures have been resolved for AE1, AE2, AE3, and BTR1 (17, 18, 43, 58, 59, 65, 66, 69, 70). The dimerization arm of the anion exchanger CTD includes the last three helices and one β-strand from the C terminus and forms a deep cleft with a characteristic V-shape, which has been used as a landmark of mutual CTD/TMD orientation in recent structural and computational papers (Fig. 1*B*) (66, 74). In addition, the dimeric interface of the CTD of the mammalian SLC4 anion exchangers remains the same regardless of the general orientation of the CTD with respect to the TMD or the presence of intracellular proteins. The CTD of BTR1 has a markedly different organization from the CTDs of the anion exchangers in which the dimerization arm is formed by β-strands, leading to a dimeric interface of predominantly β-sheet structure with a shallow cleft while the α-helical sections are positioned at the CTD periphery (Fig. 1*B*).

Most resolved SLC4 dimers feature both monomers of the TMD in the same conformational state (Table 1), either OF or IF. However, several structures of AE1, AE2, and AtBor1 were resolved in mixed states in which one monomer is in one state, while the other—in a different state. The existence of mixed monomers has been interpreted as evidence of functional independence of the monomers in the dimer (17, 48, 68, 71), which has been observed also in previous SLC4 studies (85, 86, 87) and in elevator transporters such as the aspartate transporter, Glt_Ph_ (88).

## Structural evidence for elevator transport

**Figure 3:**
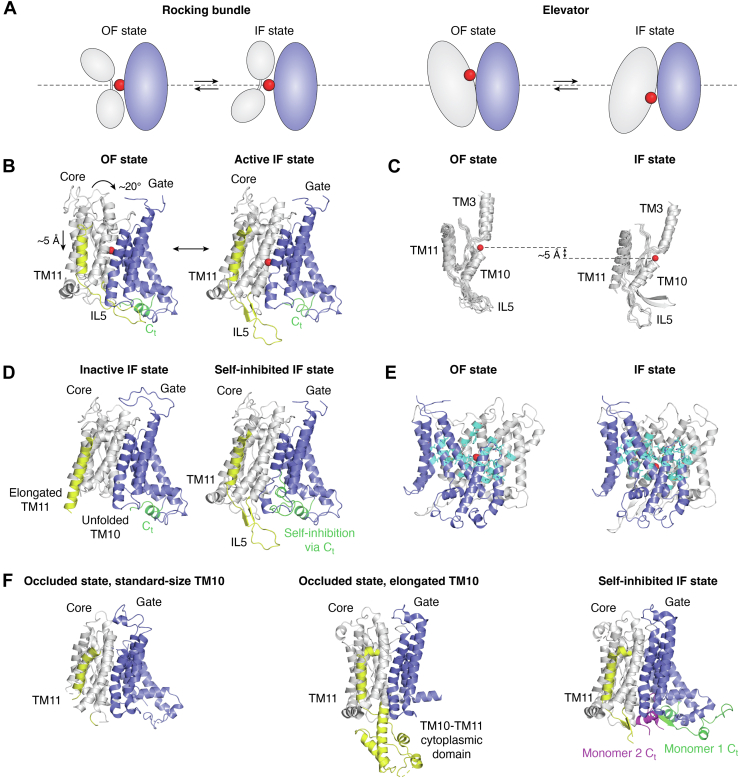
**Conformational states in the SLC4 proteins resolved by cryoEM.***A*, difference between rocking bundle and elevator transport. The rigid domain (also known as scaffold, gate) is shown as a *purple oval*. The more flexible transport domain (also known as core) is shown in *white*. The position of the binding site is indicated with a *red sphere*, while the membrane median is indicated as *dashed line*. *B*, OF ↔ IF conformational transition in AE2 (PDB ID 8GVC for OF state and 8GV9 for IF state) demonstrating the ∼20° rotation and the small ∼5 Å vertical motion of the core. The core domain is shown as *white helices*, except for TM11 and IL5 which are highlighted in *yellow*. The gate domain is presented as *purple helices*, with the C_t_ tail shown in *green*. The location of the central AE2 binding site S1 is shown as a *red sphere* at the center of mass of the AE2 S1 residues. *C*, overlap of TMs 3, 10, and 11 and IL5 from all resolved OF and IF SLC4 states displaying the ∼5 Å vertical motion of TMs 3 and 10 and the difference in TM11 length between the OF states and the putative inactive IF states. The centers of mass of the AE2 S1 residues are also shown to illustrate the magnitude and direction of the observed translocation. *D*, putative inactive *(left*, AE1 state from PDB ID 8T3U) and self-inhibited *(right*, AE2 state from PDB ID 8GVH) IF states. *E*, protein residues *(cyan sticks)* which form hydrophobic contact interfaces that occlude site S1 from the extracellular and intracellular solutions in an alternate manner during the OF ↔ IF transition. *F*, occluded (with standard-sized and elongated TM10, PDB IDs 5L25 and 8TEJ, respectively) and self-inhibited (PDB ID 8TEG) states of AtBor1. The C_t_ tails are colored *green* for the monomer shown in the figure (monomer 1) and *magenta* for the neighboring monomer (monomer 2). AE1,2: anion exchangers 1 and 2; AtBor1, borate transporter 1 in Arabidopsis thaliana; IF, inward facing state; IL5, intracellular loop 5; OF, outward facing state; SLC4, solute carrier family 4; TM, transmembrane helix; Ct, C-terminus.

Most SLC4 structures have been resolved in the OF state, with the first X-ray structure of AE1 in the OF state published in 2015 (60). An occluded structure of AtBor1 was published in 2016 (45), and presumed IF models based on low-resolution maps of Bor1p and OsBor1 were also reported in 2017 and 2021, respectively (47, 49). The first IF state of a mammalian SLC4 transporter, bovine AE1, was published in 2022 (71). Thus, in the absence of IF SLC4 structures of appropriately high resolution, early attempts of transport-type classification were made by comparison of the OF X-ray structure of AE1 with the structures of AtBor1, OsBor1, and Bor1p and existing structures of other 7+7-fold transporters in the occluded or IF state, such as the bacterial uracil/H^+^ symporter UraA, the fungal uric acid/xanthine H^+^ symporter UapA, and the bacterial fumarate/H^+^ symporter SLC26Dg (3, 45, 49, 89) or from repeat-swap modeling of AE1 (90). These studies brought forward two possible transport type options for the SLC4 mediated secondary transport—rocking bundle and elevator mechanism (Fig. 3*A*). In rocking bundle transport, the substrate binding site is positioned between a somewhat rigid and a more flexible protein domain, remains fixed within the membrane plane during the substrate translocation event, and the alternating access of the extracellular and intracellular solutions is accomplished by reorganization of the more flexible domain around the binding site with possible involvement of parts of the rigid domain in gating (91). Elevator transport also involves a binding site situated between a rigid and a flexible domain; however, the substrate translocation is accomplished by an upward and downward motion of the flexible domain with respect to the rigid domain, which results in perpendicular translocation of the binding site residues to the plane of the membrane bilayer (91, 92). To date, both OF and IF states have been published for three mammalian members of the family, the Na^+^-independent AE1, AE2, and BTR1 (Table 1), while only OF states are available for the Na⁺-dependent SLC4 transporters (the only available IF state of a Na^+^-dependent transporter, IF NBCn1, was produced by homology modeling based on an IF AE2 template). Overlap of the OF and IF structures of the different Na^+^-independent SLC4 proteins consistently reveals a small ∼5 Å vertical translocation of the central binding site (Fig. 3, *B* and *C*), which categorizes the SLC4 transport as an elevator transport mechanism. The small translocation is the result of a rigid downward motion coupled with a small (∼20°) rotation of the core with respect to the rigid gate domain, which remains largely unchanged during the OF ↔ IF transition (71). Neither the TMs from the core nor those of the gate change substantially during the conformational transition [RMSD_gate_(OF *versus* IF) and RMSD_core_(OF *versus* IF) values are ∼1 Å]. The rigid downward motion of the core is made possible by the flexible H2_TM_ and H3_TM_ arms connecting the core and gate domains and leads to small overall reorganization of the protein (and, likely, the lipid bilayer at the protein/lipid interface). The small protein and lipid reorganization during this process are in turn expected to decrease the energetic barriers for the SLC4 elevator motion, possibly influencing the high turnover rates observed in these proteins (71). Coarse-grained metadynamics calculations (71) and combined steered MD/string MD/bias-exchanged umbrella sampling simulations (68) demonstrate that the IF state of AE1 is of higher energy than the OF state, which may explain the abundance of reported OF AE1 structures relative to the IF ones. Substrate binding lowers the transition barrier and facilitates the OF ↔ IF transition (68).

The mammalian SLC4 structures resolved to date represent an OF open state and three different IF states (Fig. 3, *B*–*E*). The IF open states, such as the ones observed in AE1, AE2 and BTR1, feature a well resolved helical TM10 and can be easily related to the OF state by the rigid translocation and rotation described above (Fig. 3*B*). This structure is likely a kinetically relevant IF open state. Several bovine and human AE1 cryoEM structures feature an IF open state with partially unfolded TM10 and, interestingly, slightly elongated TM11, which has incorporated residues from IL5 (Fig. 3, *C* and *D*) (67, 68, 71). It is yet unclear if these IF states are kinetically relevant states of the SLC4 transport cycle (*e.g.*, resting or inactivated states) or if they are artifacts resulting from cryoEM sample preparation. Recent cryoEM structures of the plant SLC4 protein AtBor1 from *A. thaliana* include an IF active state with fully formed standard-sized TM10 similar to the one resolved for AE1, AE2, and BTR1 and an occluded state which demonstrates an elongated TM10 incorporating residues from the CTD-like structure between TMs 10 and 11 characteristic of plant Bor1 proteins (but absent in yeast Bor1p) (48) although such elongation was not seen in an older occluded AtBor1 intermediate resolved with X-ray (45), Figure 3*F*. A phosphorylated Thr 410 in the resolved CTD between TMs 10 and 11 of AtBor1 interacts with positively charged residues from the cytosolic side of the TMD, thus phosphorylation at this site has been suggested as a possible trigger of the conformational transitions in AtBor1. An occluded state of mammalian SLC4 has not been resolved from cryoEM or X-ray yet, and it is unclear whether elongation of TM10 or TM11 will be observed in it. Nevertheless, change in the helical and/or loop length in the TM10-IL5-TM11 region of the protein may be playing a role in the OF↔IF conformational change in mammalian and plant SLC4 transporters (48, 71).

A third type of mammalian IF SLC4 state is possibly a self-inhibited state, observed in AE2 at low pH (PDB ID 8GVH), in which the C_t_ tail of the TMD has been lodged into the IF cavity and the negatively charged terminal carboxylate group interacts with the central binding site in a similar binding configuration as the one observed for the bound substrate anions like Cl^−^ (17) (Fig. 3*D*). A self-inhibited IF state with the standard-sized short TM10, in which the IF cavity is occluded by the C_t_ tail of the neighboring monomer was also resolved for AtBor1 (48) (Fig. 3*F*). Autoinhibitory behavior *via* an unknown mechanism has been reported for the N_t_ tail in NBCe1-B and NBCe1-C isoforms (80). In human NDCBE-B/D, the C_t_ tail has inhibitory function which is not observed in human NDCBE-A/C whose C_t_ tail is significantly longer (93). The role of the N_t_ and C_t_ tail in autoinhibition thus requires further elucidation before it becomes clear how the observed self-inhibited states of AE2 and AtBor1 pertain to the SLC4 transport cycle.

A purported intermediate partially open IF AE2 structure (PDB ID: 8GVA) shows a slightly narrower IF cavity (or vestibule) compared to the open self-inhibited IF AE2 structure (17). However, it is unclear whether there is an actual mechanistic relevance of the cavity size and “openness” in these reported structures or if the larger vestibule in the AE2 self-inhibited open structure is simply a result of fitting the bulky C_t_ tail in the IF cavity. The partially open IF AE2 structure is very similar to the Cl^−^ loaded IF AE2 state (PDB ID: 8GV9, RMSD ∼0.61 Å) and the IF AE1 open state (PDB ID: 8T3U, RMSD ∼1.1 Å) (17, 67). MD simulations can elucidate questions regarding structural occlusion in published cryoEM or X-ray structures, but MD simulations have not been done with the IF AE2 structures to this end. In other members of the family, simple relaxation of the initial cryoEM or X-ray structures within the used MD simulation membranes and solutions leads to significantly higher RMSD deviations from the initial states (RMSD ∼2 Å) (62, 63, 71) than the ones obtained from overlap of the reported SLC4 open and partially open states. Thus, care should be taken when assigning single discrete states obtained from structural methods such as cryoEM and X-ray as “intermediates” since small variations due to thermal motion in the protein structure are expected for each discrete state of the transport cycle. A SLC4 state can be considered an actual intermediate occluded state, when it represents a structure in which the core assumes an intermediate position between the OF and IF state, and its central binding site is occluded from both the extracellular and intracellular solutions. No intermediate occluded states have been resolved for the SLC4 transporters, with the exception of an intermediate occluded structure obtained from computational modeling of the OF↔IF transition in NBCn1 (62) and a X-ray structure of the plant SLC4 AtBor1 transporter (45), where the occlusion of the AtBor1 transporter has not been confirmed with computational modeling. Overlap of the OF and IF NBCn1 and AE1 structures with the occluded NBCn1 and AtBor1 states, respectively, demonstrate a continuum of states consistent with the described downward motion that characterizes the SLC4-mediated elevator transport (62, 71). The modeled NBCn1 intermediate structure, which was obtained in the apo-state, possesses a hydrated substrate-binding pocket in which the water molecules are isolated from the extracellular and intracellular solution by hydrophobic contact interfaces formed by hydrophobic regions of TMs 1, 3, 8, and 10 from the core and TMs 12, 13, and 14 from the gate (Fig. 3*E*) (62). These hydrophobic contact interfaces resulting from general domain motion are also responsible for occlusion of one side of the protein in the IF and OF state.

## Identified substrate, cation, inhibitor, and lipid binding sites in the OF and IF states

Although near-atomic cryoEM maps can reveal compelling nonprotein densities, assignment of small ions (*e.g.*, Na^+^, Cl^−^, HCO_3_^−^/CO_3_^2−^) can be ambiguous because ions and ordered water molecules are difficult to distinguish, ion scattering depends on local environment, and map noise/processing can obscure weak features. Throughout this section, we therefore present binding sites identified using convergent evidence from cryoEM maps, computational modeling, functional mutagenesis, and biochemistry/transport assays, and we explicitly indicate when a density assignment remains tentative.

### Site S1 in the OF and IF states and determinants of Na^+^-independent and Na^+^-dependent transport in mammalian SLC4

**Figure 4:**
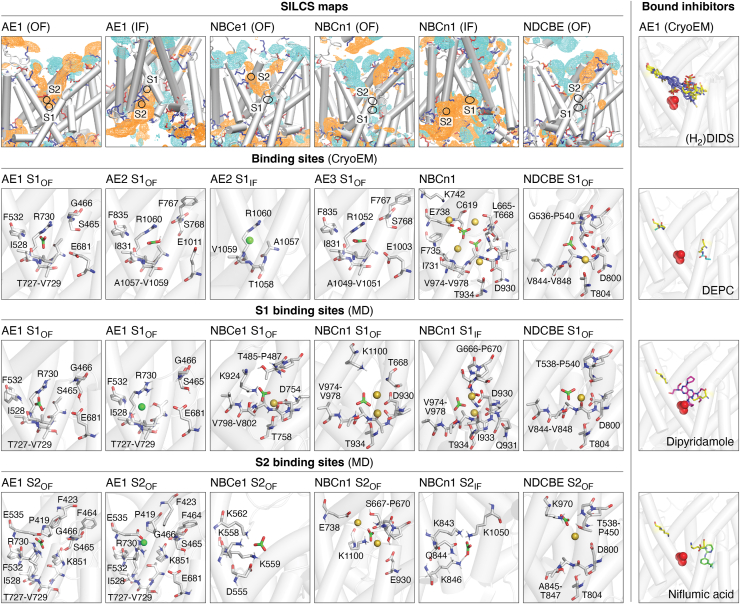
**Anionic (*orange mesh*) and cationic (*cyan mesh*) SILCS maps and experimentally (cryoEM) and computationally (MD) determined substrate, Na^+^, and inhibitor binding sites (S1 and/or S2, as discussed in the text) of various SLC4 proteins.** MD, molecular dynamics; SILCS, site identification by ligand competitive saturation; SLC4, solute carrier family 4; AE1-3, anion exchangers 1-3; NBCe1, electrogenic Na^+^-carbonate symporter 1; NBCn1, electroneutral Na^+^-carbonate symporter 1; NDCBE, Na^+^-dependent chloride/carbonate exchanger, corresponding to SLC4A8; DIDS, 4,4′-diisothiocyano-2,2′-stilbenedisulfonic acid; DEPC, diethyl pyrocarbonate.

The first TMD structures of mammalian SLC4 proteins, those of AE1 and NBCe1, were resolved without bound substrate (60, 61). The location of the binding sites at this stage was proposed to be at the protein center, in the region where the positive dipoles of the N termini of TMs 3 and 10 face each other and form a positively charged binding cavity, suitable for anion binding (3, 60, 61). Substrate binding had already been identified in this area in structures of other 7+7-fold transporters, such as UraA and UapA (94, 95). Several pathological mutations of residues in the same region have also been related to hereditary stomatocytosis in AE1 (3) and proximal renal tubular acidosis in NBCe1 (61). Cys or Ala substitution of multiple AE1 and NBCe1 residues in this area showed drastic impairment of AE1 and NBCe1 transport (72). Moreover, mutation of seven residues from the proposed binding site in NBCe1 to the corresponding residues in AE1 abolished Na^+^-dependence of transport and introduced a Cl^−^-driven base flux in this Cl^−^ independent Na^+^-CO_3_^2^^−^ symporter rendering it similar in function to AE1 and lending more evidence that this region is critical for substrate selectivity and binding in the SLC4 proteins (61). Computational modeling of OF AE1 and NBCe1 identified two putative anion binding sites in the OF cavities of these transporters: site S1 at the protein center, labeled as the “central site”, and site S2 found closer to the OF cavity entrance, labeled as the “entry site” (Fig. 4) (68, 72). Based on the ion dynamics observed during MD simulations and previous electrophysiology studies (96, 97), it was suggested that the entry sites are involved in selective attraction of ions from the surrounding solution and successive ion redirection toward or away from the central site, which is the main catalytically relevant site of the SLC4 proteins (72). Substrate binding at the central site has been hypothesized as the required event triggering the conformational transition between the OF and IF states, although the existing biased and unbiased MD simulations have so far been unable to demonstrate how this triggering occurs. More recent cryoEM structures of OF NDCBE, NBCn1, AE1, and AE2 with bound substrates (17, 62, 63, 64, 67) confirmed the computationally identified central binding sites S1. Several substrate-bound structures of IF AE2 were also resolved showing for the first time the structural changes in site S1 between the OF and IF conformations of a Na^+^-independent SLC4 transporter (Fig. 4) (17).

Generally, with the exception of BTR1, the region of site S1 in the mammalian SLC4 family features two major anion-binding configurations, which can be distinguished based on their amino acid composition and which correspond to the Na^+^-independent anion exchangers (AE1, AE2, and AE3) and the Na^+^-dependent symporters and exchangers of the family (NBCn1, NBCn2, NDCBE, NBCe1, and NBCe2) (Fig. 2, Table 2). In this respect, the analogous site S1 of the AE4 transporter (if AE4 has the same 7+7-fold structural organization as the other members of the family) more closely resembles the binding site of the Na^+^-dependent symporters and exchangers, according to recent modeling and functional mutagenesis data (77) and in line with its suggested function as a cation-dependent CO_3_^2^^−^/Cl^−^ exchanger (11). Within these two configurations, sequence identity in site S1 is very high (Fig. 2). The most significant difference between the subgroups of the Na^+^-independent and Na^+^-dependent transporters appears to be the existence of an Arg residue on TM10 in site S1 of the anion exchangers AE1-AE3, which is absent in the Na^+^-dependent SLC4 members and AE4, and a central Glu residue on TM8 in AE1-AE3, which is substituted by an Asp residue in the Na^+^-dependent SLC4 members and AE4. The central sites S1 of several SLC4 transporters extracted either from the reported cryoEM structures or from MD simulations are shown in Figure 4. In cryoEM structures of the Na^+^-independent anion exchangers of the family (AE1-AE3) bound to HCO_3_^−^, the anion is bound by the headgroup of the central Arg residue and stabilized further at the N terminus of TM10 by the backbone -NH groups of the Arg and the residues preceding the Arg (17, 64, 67, 68, 69). In AE1, which features two Thr residues in site S1, the second Thr residue (T727) is involved in anion coordination *via* its -OH group. This binding pattern is reproduced also by MD simulations (Fig. 4). In addition, MD simulations in AE1 with HCO_3_^−^ and Cl^−^ bound to site S1 (68, 72) show identical binding patterns for both anions despite their different sizes and geometry (Fig. 4). Only one cryoEM OF structure of AE1 bound to Cl^−^ is available (67). In this structure, the Cl^−^ ion has shifted slightly from the central Arg toward the central Glu residue but is still in the coordination pocket of site S1 and is coordinated mainly by electrostatic interactions with the central Arg. In OF AE1 structures bound to HCO_3_^−^ interaction energy calculations demonstrate that HCO_3_^−^ does not participate in significant interactions with the residues of TM3, while simulated annealing of chemical potential calculations yield a K_d_ value for HCO_3_^−^ in site S1 of 1.5 mM in good agreement with the experimentally measured 2.0 to 5.4 mM (64). Higher K_d_ values have been evaluated from MD interaction analysis for HCO_3_^−^ (22 mM *versus* 38 mM) and Cl^−^ (255 mM *versus* 364 mM) in OF *versus* IF AE1 state (68).

**Table 2:** Protein residues involved in ion binding in the SLC4 proteins identified from structural and computational studies

System	Binding site (method)	Binding site composition	Ref.
hAE1	S1_OF_ (cryoEM)	**TM3:** S465, G466; **TM5:** I528, F532; **TM8:** E681; **TM10:** T727-V729, R730	(64)
	S1_OF_ (MD)	**TM3:** S465, G466; **TM5:** I528, F532; **TM8:** E681; **TM10:** T727-V729, R730	(72)
	S2_OF_ (MD)	**TM1:** P419, F423; **TM3:** F464, S465, G466; **TM5:** I528, F532, E535; **TM8:** E681; **TM10:** T727-V729, R730**, TM13:** K851	(72)
hAE2	S1_OF_ (cryoEM)	**TM3:** F767, S768; **TM5:** I831, F835; **TM8:** E1011; **TM10:** A1057-V1059, R1060	(17)
	S1_IF_ (cryoEM)	**TM10:** A1057-V1059, R1060	(17)
hAE3	S1_OF_ (cryoEM)	**TM3:** F767, S768; **TM5:** I831, F835; **TM8:** E1003; **TM10:** A1049-V1051, R1052	(69)
hAE4	S1_OF_ (MD)	**TM3:** T448, G449, P450; **TM8:** D709, T713 **TM10:** T756; **TМ13:** K879	(77)
hNBCe1	S1_OF_ (MD)	**TM3:** T485-P487; **TM8:** D754, T758; **TM10:** V798-V802	(72)
	S2_OF_ (MD)	**TM5:** D555, K558, K559, K562	(72)
hNBCn1	S1_OF_ + S2_OF_ (cryoEM)	**TM1:** C619; **TM3:** L665-T668; **TM5:** I731, F735, E738, K742; **TM8:** D930, T934; **TM10:** V974-V978	(62)
	S1_OF_ (MD)	**TM3:** T668; **TM8:** D930, T934; **TM10:** V974-V978; **TМ13:** К1100	(62)
	S2_OF_ (MD)	**TM3:** S667-P670; **TM5:** E738; **TM8:** D930; **TМ13:** К1100	(62)
	S1_IF_ (MD)	**TM3:** G666-P670; **TM8:** D930, Q931, I933, T934; **TM10:** V974-V978	(62)
	S2_IF_ (MD)	**TM5:** K843, Q844, K846; **TM12**: K1050	(62)
rNDCBE	S1_OF_ (cryoEM)	**TM3:** G536-P540; **TM8:** D800, T804; **TM10:** V844-V848	(63)
	S1_OF_ (MD)	**TM3:** T538-P540; **TM8:** D800, T804; **TM10:** V844-V848	(63)
	S2_OF_ (MD)	**TM3:** T538-P540; **TM8:** D800, T804; **TM10:** A845-T847, **TM13:** K970	(63)

Residues identified solely from functional mutagenesis are not reported in Table 2.

In the IF states of AE2 which were resolved with bound Cl^−^ (17, 18), the binding pattern for Cl^−^ resembles that for HCO_3_^−^ and Cl^−^ bound to OF AE1. Overlap of the residues of site S1 between the OF AE2 bound to HCO_3_^−^ (PDB ID: 8GVC) and IF AE2 bound to Cl^−^ (PDB ID: 8GV9) conformations yields RMSD ∼0.36 Å which shows an almost identical organization of the S1 residues with the small variation likely due to the different size and shape of the bound anion. This is consistent with the elevator transport mechanism in the SLC4 family which involves a rigid motion of the core (with its bound substrates) with respect to the gate, with very little protein reorganization (62, 71).

There is an ongoing debate whether the Na^+^-dependent SLC4 transporters transport bicarbonate or carbonate and what number of Na^+^ ions are transported together with the anionic substrate (2, 14, 63, 72, 98). Several recent studies on NBCe1 reveal that its likely anion substrate is a carbonate rather than a bicarbonate ion, leading to a Na^+^-CO_3_^2^^−^ transport stoichiometry for electrogenic transport of charge −1, as observed during the import of a Na^+^ and a CO_3_^2^^−^ ion in a variety of cells, including astrocytes, pancreatic duct cells, myocytes, *Xenopus* oocytes, and HEK293 cells (1, 14). This conclusion is supported also by cryoEM structures and modeling studies of NDCBE and NBCn1 that show that the most likely substrate stoichiometries are a Na^+^-CO_3_^2^^−^ ion pair in the OF Na^+^-dependent CO_3_^2^^−^/Cl^−^ electroneutral exchanger NDCBE (63) and a 2Na^+^-CO_3_^2^^−^ ion triplet in the OF electroneutral Na^+^-dependent CO_3_^2^^−^ transporter NBCn1 (62). Whether bicarbonate or carbonate is transported by NBCe2 and NBCn2 has not been determined; however, considering the sequence similarities of the TMD domains in the Na^+^-dependent SLC4 transporters (Fig. 2), they are likely the same as NBCe1 and NBCn1, respectively. Despite its low concentration at physiological pH ∼7.4 (extracellular) and 7.15 (intracellular), for a given transport event, carbonate transport is more efficient due to its ability to bind two protons, leading to more effective base loading or extrusion (62).

Although the order of Na^+^ and CO_3_^2^^−^ binding has not been determined, computational modeling of OF NBCe1 (72), later confirmed by a cryoEM structure and modeling of Na^+^-CO_3_^2^^−^-bound OF NDCBE (63), revealed that Na^+^ and CO_3_^2^^−^ exist as an ion pair in the almost identical central sites S1 of OF NBCe1 and OF NDCBE and that the Na^+^ ion, bound at the central Asp residue on TM8, serves as an anchor of the CO_3_^2^^−^ anion in the binding pocket in the absence of a positively charged residue like the central Arg in the AE1-AE3 anion exchangers. Additional stabilization of the substrate anion in the binding pocket was achieved *via* interaction with the backbone -NH groups of a stretch of hydrophobic residues from TM10 and the anion and cation interactions with the hydroxyl groups of Thr residues in TM8 and TM10 (Table 2, Fig. 4). Residues at the N terminus of TM3 are also involved in ion coordination (Table 2, Fig. 4). Free energy of binding simulations of different ion combinations in OF NDCBE provided additional evidence of the anchoring role of the Na^+^ bound at the central Asp residue of TM8 and identified the preferred ion binding stoichiometry for site S1 as a Na^+^-CO_3_^2^^−^ ion pair. A recent NBCn1 OF structure features 4Na^+^ and 2CO_3_^2^^−^ ions in the binding pocket of site S1, which is large enough to accommodate these ions (Fig. 4). However, considering the difficulties in distinguishing water and ions during the assignment of cryoEM densities, MD simulations were also done for better resolution of the ion binding sites in NBCn1 and suggest that the transport relevant ion stoichiometry of NBCn1 is 2Na^+^-CO_3_^2^^−^ instead of 4Na^+^-2CO_3_^2^^−^. In this case, both Na^+^ ions are coordinated by the Asp and Thr residues of TM8 with the two Na^+^ cations and the CO_3_^2^^−^ anion effectively bound as an ion triplet (62). The remaining anion and cation coordination is similar as the ones observed in NBCe1 and NDCBE (Table 2). A nearby Lys residue from TM13 is also involved in occasional anion coordination in site S1 in the studied Na^+^-dependent SLC4 transporters, although it is a more prominent coordinating residue in site S2 (see below).

A recently reported homology model of AE4 based on the OF NDCBE cryoEM structure identifies a putative ion binding site in the same area as site S1 in NBCe1, NDCBE, and NBCn1, with mutation of key residues from TM3, TM8, and TM10 impacting alkalinization rates both in the presence of external Na^+^ and K^+^ (77). MD simulations of WT and mutant AE4 loaded with Na^+^-HCO_3_^−^ or K^+^-HCO_3_^−^ ion pairs confirm Na^+^ or K^+^ coordination at the Asp and Thr from TM8 and participation of the Lys residue of TM13 in anion coordination analogous to the cation and anion binding in the other studied Na^+^-dependent members of the family (62, 63, 72).

The central Asp residue from TM8 therefore emerges as a critical determinant of cation-dependent transport, considering its involvement in Na^+^ coordination in the identified sites S1 of NBCe1, NDCBE, and NBCn1, Na^+^ and K^+^ coordination in site S1 of AE4, and its high conservation in all cation-dependent members of the family (including AE4, Fig. 2) (61, 62, 63, 72, 77). In the anion exchangers AE1-AE3 and BTR1, instead, the analogous central acidic residue is a Glu (Fig. 2). Both transport in the Na^+^-independent anion exchangers and transport in the Na^+^-dependent members of the family is sensitive to mutations at the central acidic residue at site S1 (61, 62, 63, 72, 77). Cys substitution of this central Glu residue leads to an effectively inactive AE1 transporter. Substitution of Cys in place of the central Asp in NBCe1 inactivates NBCe1 transport, while the charge-preserving Asp to Glu substitution leads to severe transport impairment. The shorter side chain of Asp in the Na^+^-dependent members of the family may be required to fit one or more Na^+^ ions, together with the substrate anion(s) in site S1. In addition, Asp has lower pKa value than Glu (pH 3.5 *versus* 4.2, respectively) (99) which makes its protonation less probable and guarantees that the negative charge at the central site S1 remains ready for Na^+^ coordination (72). The role of the central Glu in the anion exchangers and BTR1 is less clear. The central Glu residue in AE1 has been identified as a H^+^ binding site during H^+^-SO_4_^2^^−^ cotransport (100) and a Glu to Gln substitution converts AE1 into a pH-independent electrogenic SO_4_^2^^−^/Cl^−^ exchanger (101). Whether protonation of this Glu (at the physiological pH ∼7.2–7.4) is an important part of the transport function of the anion exchangers (*e.g.*, as a proton sensor) remains to be seen. Propka simulations have shown a pKa value of 6.8 for the central Glu residue in OF AE1, hinting that this residue might protonate/deprotonate easily in response to environmental and structural factors (72). MD simulations with protonated central Glu lead to more frequent entry of Cl^−^ ions from the extracellular solution, longer Cl^−^ residence times in the vicinity of site S1, and frequent presence of more than one Cl^−^ ion in the site S1-binding pocket (72). This can in turn facilitate the binding and transport of Cl^−^ (and, likely, HCO_3_^−^) by AE1.

NBCe1 has been hypothesized to transport two net negative charges, *e.g.*, Na^+^-CO_3_^2^^−^-HCO_3_^−^, both *in vivo* and in a conformational state-dependent manner (14). It is unclear (from measured NBCe1 function of heterologously expressed protein that demonstrates transport of 1 net negative charge through the transporter, coupled with insights from structural OF data) how a Na^+^-CO_3_^2^^−^-HCO_3_^−^ transport stoichiometry is achieved. Moreover, in the absence of IF structures of Na^+^-dependent SLC4 transporters, accurate computational assessment of their IF central binding sites and whether the IF state can accommodate two anions is not possible. It remains to be seen if site S1 in the Na^+^-dependent SLC4 proteins undergoes negligible changes between the OF and IF conformations, as observed in the anion exchanger AE2. IF models of NBCe1 and NBCn1 based on the IF AE2 structure (PDB ID: 8GV9) show that the geometry of site S1 undergoes a small change during the OF to IF transition (62, 102), and the ion triplet in NBCn1 remains bound in a similar binding pattern as the one observed in the OF state (62). The small change can be summarized as loss of interaction with gate residues prominent in the OF state and in proximity of the OF site S1 and instead enhanced coordination with the residues of TM3 (Fig. 4, IF NBCn1). However, modeling of ion-bound IF states in NBCe1 has not been done, and putative sites for an additional HCO_3_^−^ ion have not been discussed. The IF anion exchanger template might also be inappropriate for the modeling of an additional HCO_3_^−^ site.

The last member of the SLC4 family, BTR1, which has the lowest sequence similarity with the remaining SLC4 proteins (Fig. 2), is also quite different in terms of transport properties (12, 13). It was initially thought that BTR1 transports Na^+^-B(OH)_4_^−^, but subsequent studies have reported that BTR1 mediates H^+^/OH^−^ transport, NH_3_-H^+^ symport, or allosteric NH_3_/NH_4_ activation of H^+^/OH^−^ transport. The reported cryoEM OF BTR1 structure does not provide assignment of NH_3_ binding sites but reports two small cryoEM densities in the vicinity of the residues corresponding to site S1 in the remaining SLC4 members (43). These densities have been proposed to be either NH_4_^+^ ions or water molecules, but definitive assignment has not been made. The amino acid composition of site S1 in BTR1 has some unique features, such as the existence of two His residues in the beginning of TM10 and lack of Thr residues in TM8. In the area of the site S1 residues of TM3, BTR1 bears some resemblance with the Na^+^-dependent transporters of the family (LTTAP *versus* GSTGP motif, Fig. 2). The two His residues of site S1 are obvious options for putative H^+^ binding sites, considering that the pKa value of His (pKa ∼6.6) (99) is close to the physiological intracellular pH of 7.15. However, it is currently unclear if NH_3_ can bind in the same S1 site and how the overall NH_3_-H^+^ substrate binds and is transported. It has also been proposed that BTR1 transports H^+^/OH^−^, but the exact binding sites and potential modulation by NH_3_/NH_4_ in reaction with the transporter is unknown. Nevertheless, the central area of BTR1 that corresponds to site S1 in the remaining SLC4 transporters could be able to accommodate also anions, especially if the central His (corresponding to the central Arg in AE1-AE3) is protonated, following similar binding patterns as the ones observed in AE1-AE3. Anion permeation in the OF cavity should also be trivial in BTR1, considering the three Lys residues lining TM5, leading to the protein center (K508, K512, and K516) (Fig. 2).

Substrate binding sites for borate in the plant and yeast Bor1 transporters have not been resolved structurally or computationally yet. However, functional mutagenesis studies, complementation yeast assays, and *in vivo* plant phenotype analysis for various mutants of AtBor1 have singled out a number of residues from the protein center corresponding to site S1 in the mammalian SLC4 proteins. Among them is a central Asp residue on TM8, analogous to the central Asp/Glu in mammalian SLC4, which is the likely H^+^ binding site in AtBor1 (45, 47, 48).

### Permeation pathways and entry sites S2 in the OF and IF states

So far, there are no published structures of SLC4 proteins with bound ions at the computationally predicted entry sites S2. The identification of these peripheral sites in a few SLC4 members (AE1, NBCe1, NDCBE, and NBCn1) has been achieved entirely through computational modeling by cross-referencing results from site identification by ligand competitive saturation (SILCS) calculations (with acetate and methylammonium probes used as surrogates for anions and cations, respectively) and unbiased MD simulations with the physiological ions of importance (*i.e.* Na^+^, Cl^−^, HCO_3_^−^, CO_3_^2^^−^) (62, 63, 68, 71, 72). Generally, the agreement of the SILCS cationic and anionic maps with simulated cationic and anionic density maps obtained from MD has been excellent (62, 63, 71, 72). Comparison of the SILCS maps of the OF AE1, NBCe1, NDCBE, and NBCn1 (Fig. 4) shows some interesting similarities and differences. In the OF AE1 and NBCe1, cations do not permeate the large well hydrated OF cavity to the protein center, even though a small cationic density is visible in the vicinity of the central Asp, which is the main Na^+^ binding residue of site S1 in NBCe1 (Fig. 4). Anion entry in the OF cavity, however, is easy and occurs along the portions of both TM5 and TM3 that are in contact with the extracellular solution. MD simulations confirm the SILCS findings with multiple anion entries in the course of the MD simulations and substrate anions frequently reaching the protein center and the binding cavity of site S1 of OF AE1 (68, 72). The other two Na^+^-dependent SLC4 members, NDCBE and NBCn1, also feature a small cation density in their SILCS maps at the central Asp residue of site S1; however, they have clear well delineated pathways for cations (along TM5) and anions (along TM3) which demonstrate ease of access of the Na^+^ ions in the OF cavity. MD simulations confirmed the SILCS cationic and anionic pathways and demonstrated entry of both Na^+^ and anions in the OF cavity of NDCBE and NBCn1 (62, 63). MD simulations with position restraints imposed on the CO_3_^2^^−^ ion were used to assess the energetics of permeation in OF NBCn1 and demonstrated that the anion can permeate vertically in the OF cavity along a pathway featuring several small energy minima and energetic barriers of 1 to 2 kcal/mol (62).

The anion entry sites S2 in the OF state lie on the anion permeation pathway and themselves are part of the pathways (Fig. 4). In the OF AE1, anions freely traverse the cavity along the observed anion pathway toward the protein center and frequently bind to site S2, which is formed by key residues of TM3, TM5, TM10, and TM13 (Fig. 4, Table 2) (72). Site S2 is positioned just above site S1 in the OF cavity of AE1. The long side chain of the central Arg on TM10 participates in ion binding both in sites S1 and S2 and is the major driver for anion transition from the entry site S2 to the central site S1 (72). In the Na^+^-dependent members of the family, NDCBE and NBCn1, site S2 is found just above site S1 (similar to the AE1 case), at the bottom of the ion permeation pathways (62, 63). However, due to the absence of a central positively charged residue, the coordination relies mostly on a Lys residue from TM13 and residues from TM3, Figure 4, Table 2. It is currently unknown whether an anion or a cation binds first to site S2. However, the presence of a bound ion in site S2, regardless of its charge, tends to draw the counter ion from the surrounding solution (62, 63). MD simulations of NDCBE demonstrated motion of the bound Na^+^-CO_3_^2^^−^ pair between sites S1 and S2, similarly to the anion motion between sites S1 and S2 in AE1, lending additional evidence for the potential role of site S2 in selection and attraction of ions from the external solution and their redirection toward site S1. This is in line with previous electrophysiology results in AE1 that indicate existence of more than one binding site with different functional roles (*i.e.,* approach and alternating site, which correspond to the entry/S2 and central/S1 sites, respectively) (96, 97). Regardless, the existence of sites S2 and their specific role during the SLC4-mediated transport processes require further elucidation.

The Na^+^-dependent NBCe1 shows some marked differences from the ion permeation and binding trends demonstrated by MD simulations in AE1, NBCn1, and NDCBE. Site S2 in NBCe1 was identified at the top of the OF cavity, at a cluster of Lys residues of TM5 far away from the central site S1 (Table 2, Fig. 4). The SILCS and MD simulations of NBCe1 could not identify a Na^+^ permeation pathway to site S1. Instead, the three Lys residues at the cavity entry seem to act as an electrostatic barrier preventing Na^+^ entry into the cavity. Negatively charged anions, such as CO_3_^2^^−^, HCO_3_^−^, and Cl^−^, form long-lasting interactions with this positively charged Lys cluster. Once an anion is bound at site S2 in NBCe1, Na^+^ ions start permeating deeper into the cavity in the direction of the negatively charged residues of TM5 and the central site S1 with its Asp residue. Although no full Na^+^-CO_3_^2^^−^ permeation and binding event was observed in the MD simulations of OF NBCe1, it is likely that shielding the Lys cluster by anion binding is a prerequisite for Na^+^ permeation in the OF cavity (72) leading to co-operative cation–anion permeation and binding of unclear mechanistic significance. Although no specific computational or modeling studies have been done for assessment of the role of the residues of site S2 in ion selectivity, some evidence of their functional importance for substrate selectivity has been found in the NBCe1 D555E substitution (103, 104). Residue D555 is part of site S2 in NBCe1 and a Glu substitution in this position induces a Cl^−^ flux which does not exist in WT NBCe1. Cl^−^ transport in the absence of bicarbonate was significantly greater than in the presence of bicarbonate buffer when Na^+^ was also present suggesting that Cl^−^ competes with Na^+^-CO_3_^2^^−^ binding. Whether a new channel is formed in the mutant or Cl^−^ interaction with site S1 can induce an OF↔IF conformational change (uniporter transport mode) is unknown.

The ion dynamics and permeation in the OF state are almost completely controlled by the TM3 and TM5 portions exposed to the extracellular solution. Sequence comparison in this area shows significantly lower sequence identity than the one observed in the area of the central binding sites S1 even within the subgroups of Na^+^-dependent and Na^+^-independent members of the family (Fig. 2). In the Na^+^-dependent members of the family, the extracellular portion of TM3 generally contains more positively charged residues and appears as the preferred anion pathway in SILCS and MD simulations of NBCn1, NBCe1, and NDCBE (Fig. 4). TM5 in the Na^+^-dependent SLC4 transporters however offers a variety of amino acid patterns that can lead to fine-tuned ion permeation in the OF cavity: the electroneutral transporters NBCn1 and NBCn2 feature 3 negative and 1 positive residue, leading to predominantly cation permeation (as seen in simulations of NBCn1), the electrogenic transporters NBCe1 and NBCe2 have 2 to 3 positive residues and 1 negative, leading to anion entry (as seen in simulations of NBCe1), and the sodium dependent anion exchanger NDCBE has 2 negative and 1 positive residue, leading to similar permeation frequencies for cations and anions. In the anion exchangers of the family, TM3 has few charged residues, and they differ between AE1-3. SILCS and MD simulations of human and bovine AE1 show predominant anion permeation in this area regardless of the presence of negatively charged residues (71, 72), likely due to the latter orientation away from the ion permeation path. TM5 in the exchangers feature 2 positive and 1 negative residue and lead to anion permeation in the OF cavity as observed in simulations of AE1 (Fig. 4).

The different permeation patterns observed in the MD simulations of OF AE1 and the Na^+^-dependent members of the family NBCe1, NDCBE, and NBCn1 may have an impact on the overall transport rate considering that ion permeation to and from the central binding sites represent steps in the SLC4 transport cycle. Turnover rate has been measured for kidney and erythrocyte AE1 (∼50,000–75,000 s^−1^ transport events per second), NBCe1 (∼30,000 s^−1^), and NBCn1 (∼15,000 s^−1^) (62, 96, 102) showing remarkably fast transport in all three members of the family. If AE1, NBCn1, and NBCe1 undergo similar conformational transformation with similar rates of the OF↔IF state transition, it is possible that the significantly lower transport rates of NBCe1 and NBCn1 could be a result of slower ion permeation due to the absence of the central Arg residue which swiftly draws in the substrate anions to the central site S1. An additional consideration that can explain the difference in transport rates is the concerted permeation of a different number of permeating ions (1 in AE1 *versus* 2 in NBCe1 *versus* 3 in NBCn1) or the existence of strong electrostatic barriers at the cavity entry, such as the Lys cluster observed at site S2 in NBCe1 that necessitate cooperative cation–anion entry in the OF cavity.

Permeation pathways have also been assessed with SILCS and MD simulations of the IF states of AE1 and NBCn1 (62, 68, 71). In both cases, large anion densities, indicative of the existence of anionic reservoirs, are present at the intracellular side of the protein in the area of the dimeric interface formed by gate helices TMs 6 and 7 (62, 71). The anions from these reservoirs can then move laterally toward the IF cavity and the central site S1. Unlike the more nuanced differences in ion permeation in the OF states, the same anion entry patterns are observed in both IF AE1 and IF NBCn1 SILCS and MD simulations. This is supported by the high sequence similarity of the SLC4 proteins observed in these areas of TMs 6 and 7 (Fig. 2). An IF site S2 (an analog of the sites S2 in the OF states of AE1, NBCe1, NBCn1, and NDCBE) has been computationally identified only in an IF homology model of NBCn1 (Fig. 4) in the area of the anionic reservoir, at a cluster of positively charged residues at the intracellular ends of TMs 6 and 7 and the intracellular loop between them (62). Residues from this area also have frequent interactions with anions in MD simulations of IF human AE1, although specific anion binding sites were not reported in these studies (68). This site is lying on the anion permeation pathway into the IF cavity of NBCn1 and is frequently occupied by an anion during MD simulations. Anionic density in this area of TMs 6 and 7 is also present on ion density maps in IF bovine AE1 (71) and even in the simulations of the OF states of AE1, NBCe1, NDCBE, and NBCn1 (62, 63, 72), where the IF cavity is occluded by the upward motion of TM10. Presence of a prominent anion binding site in this area could be potentially useful both for facilitating anion entry from the reservoir toward the central site S1 (when anions are transported from the intracellular side to the extracellular side) and for facilitating the exit of anions bound at site S1 (in the OF to IF direction), depending on electrochemical gradients and physiological direction of transport.

### Binding of inhibitors

A number of AE1, AE2, and AE3 structures have been resolved in the OF state with bound inhibitors, including DIDS (4,4′-diisothiocyano-2,2′-stilbenedisulfonic acid), H_2_DIDS (4,4′-diisothiocyano-dihydrostilbene-2,2′-disulfonic acid), diethyl pyrocarbonate (DEPC), dipyridamole, and niflumic acid (Fig. 4) (17, 60, 64, 69). DIDS and H_2_DIDS belong to the stilbene disulfonic acid group of nonspecific inhibitors of the SLC4 family and inhibit most of the members (4). The first TMD structure of a SLC4 protein, the X-ray structure of AE1 was resolved with bound H_2_DIDS and provided indication for covalent crosslinking of H_2_DIDS with both K539 of TM5 and K851 of TM13 (60). More recent structures of AE1, AE2, and AE3 bound to DIDS or H_2_DIDS demonstrate covalent binding only at the Lys of TM5 analogous to K539 in AE1 and ionic interaction of the sulfonic acid group of stilbene with the Lys on TMs 13 and polar residues in its vicinity on TM13 and 14 (17, 64, 69). DEPC was initially thought to inhibit the IF state of AE1 *via* covalent binding to H834 in the beginning of TM13 (105, 106); however, an AE1 structure with two covalently bound DEPC molecules at K539 and K851 was resolved recently in the OF state (64). Unlike DIDS, H_2_DIDS, and DEPC, which interrupt anion exchange due to competitive inhibition with the anionic substrate or disruption of the anionic binding site, dipyridamole presumably blocks AE1 transport by blocking the substrate permeation (107). It occupies the same space in the OF cavity as DIDS and H_2_DIDS; however, given its lack of negatively charged groups, it is not expected to interfere with anion binding. A recent OF AE3 structure that was resolved with both DIDS and HCO_3_^−^ bound at the same time indicates that binding of anions to the central site S1 is possible even in the presence of DIDS and H_2_DIDS, at least in AE3 (69). Niflumic acid does not affect substrate affinity or access to the binding sites. The relevant OF AE1 + niflumic acid structure and docking studies reveal a slightly different binding site in the OF cavity for niflumic acid, involving TMs 8, 13, and 14, where it could impact the OF↔IF conformational transition (64, 108).

Generally, most inhibitors, except niflumic acid, bind in similar areas in the OF cavity which overlap with the bottom of the ion permeation pathways and the location of sites S2 in AE1, NBCn1, and NDCBE (Fig. 4). The location of the inhibitor in the resolved structures and the involved residues from site S2 and the permeation pathways could explain both the competitive inhibition and the permeation obstruction.

### Binding of lipids

Most of the experimental evidence for lipid effects on SLC4 function comes from studies on AE1, NBCe1, and NBCn1 and as such the protein–lipid interactions that underpin the potential lipid modulation of the transport by the entire SLC4 family are still unclear (76). AE1 interacts specifically with cholesterol (CHOL) (109, 110), and these interactions depend on the surrounding lipids (111, 112). Specific PIP_2_-AE1 interactions have also been observed (113). Anion transport in AE1 is inhibited by sphingomyelin and phosphatidylserine (PS) but unaffected by phosphatidylethanolamine (114). Most lipid effects on AE1 function have been proposed to stem from rigidification of the membrane due to saturated lipid tails, formation of lipid–protein H-bonds, electrostatic interactions between the anions and the negative lipid heads, and lipid-dependent aggregation of hAE1 into dimers and tetramers (76). Cytosolic PIP_2_ stimulates different isoforms of NBCe1 (-A, -B, and -C) to a different extent and according to two different mechanisms, indirectly *via* hydrolysis of PIP_2_ to IP_3_ (in the B and C isoforms) and directly (in the -A isoform) (80, 115, 116, 117). A putative PIP_2_ binding site, which is also required for modulation by inositol 1,4,5-trisphosphate binding protein in NBCn1-A and NBCe1-B, has been identified in the autoinhibitory domain of the NBCe1-B N_t_ tail (residues 37–65) (80).

**Figure 5:**
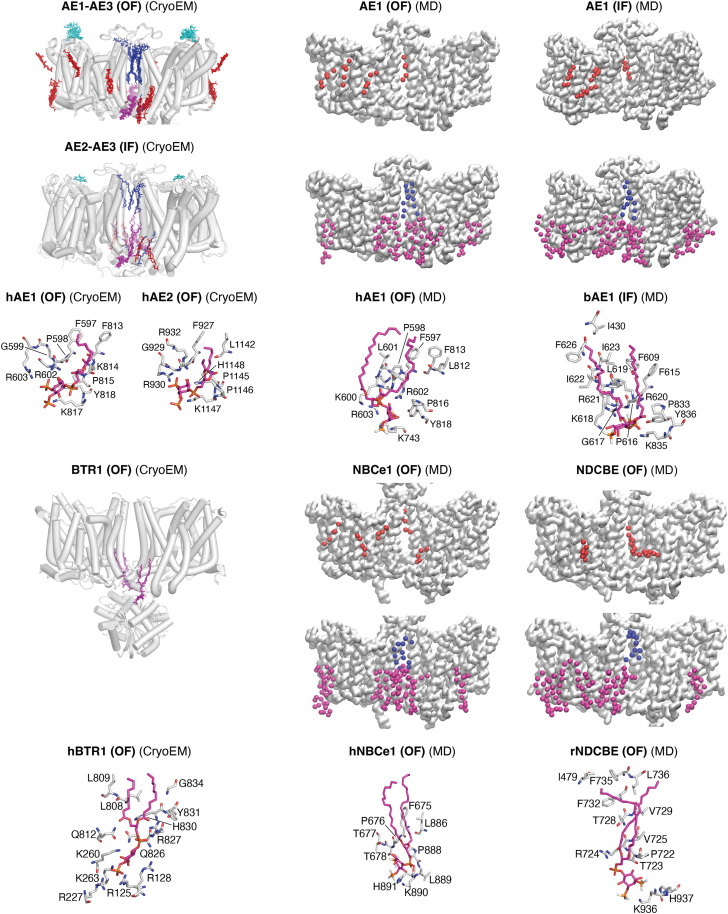
**Experimentally and computationally resolved lipid binding sites in various SLC4 proteins.** The protein matrix is presented either as *white cylinders* or *white sticks* (in all-atom representation from cryoEM structures or all-atom MD simulations) or *white surface* (in coarse-grained representation from CGMD simulations). Cholesterol/cholesterol derivatives and phospholipids are shown as *red* or *blue sticks* (all-atom representation from cryoEM structures) or *red* or *blue spheres* (coarse-grained representation from CGMD simulations). PIP_2_/PIP_2_ derivatives are shown as *magenta sticks* (all-atom representation from cryoEM or all-atom MD simulations) or *magenta spheres* (coarse-grained representation from CGMD simulations). The N-linked glycosylation sites resolved by cryoEM in AE1-AE3 are shown as *cyan sticks*. AE1-3, anion exchangers 1, 2, and 3; CGMD, coarse-grained MD; MD, molecular dynamics; PIP_2_, phosphatidylinositol bisphosphate; NBCe1, electroneutral Na^+^-carbonate symporters 1 and 2, corresponding to SLC4A7 and SLC4A10, respectively; NDCBE, Na^+^-dependent chloride/carbonate exchanger, corresponding to SLC4A8; BTR1, a human SLC4 transporter, corresponding to SLC4A11; OF, outward facing state, IF, inward facing state.

Recent SLC4 structures with bound CHOL, CHOL derivatives, phospholipids, and/or PIP_2_-like lipids have so far been resolved for AE1 (OF and IF states) (64, 65, 67, 68), AE2 (IF state) (17, 18), AE2(TMD)-AE3(CTD) chimera (IF state) (69), and BTR1 (OF state) (43, 70), Figure 5. A binding site for a PIP_2_-like lipid is consistently found at the TM6 of one monomer and H4_TM_ and H5_TM_ of the second monomer, at the dimerization interface of the TMD in multiple AE1 and AE2 structures and in the AE2(TMD)-AE3(CTD) chimera, regardless of CTD presence and TMD conformation state. In AE2, it has been hypothesized that PIP_2_ binding leads to dissociation of the TMD and CTD, which results in cryoEM structures of the TMD without resolved CTD (18). In BTR1, a PIP_2_ binding site is resolved at the CTD/TMD (TM13 and H5_TM_) at a slightly shifted position from the PIP_2_ site observed in the other members of the family (43, 70). Low pH or an R125H mutation lead to loss of PIP_2_ binding, 180° rotation of the CTD domain, and strong association between the CTD and TMD which presumably locks the BTR1 structure in the IF state. Recently reported 14 cryoEM maps of BTR1 at different pH and different salt conditions (KCl, NaCl, NH_4_Cl, or NaCl + NH_4_Cl mixtures) however capture both OF and IF states of WT BTR1 at acidic, neutral, and basic pH which shows that the PIP_2_ binding and pH effects might not be imperative for the conformational transition in BTR1 (70). Other than the cryoEM structures with/without resolved PIP_2_, conclusive evidence about the effect of PIP_2_ on the CTD/TMD association and TMD conformation state in AE2 and BTR1 (and other members of the family) has yet to be produced. In addition, since unambiguous functional evidence of PIP_2_ regulation has been demonstrated only for AE1, NBCe1, and NBCn1 (68, 80, 115, 116, 117), it is unclear whether the resolved PIP_2_ binding sites in AE2, AE3, and BTR1 are actually involved in PIP_2_ regulation in these proteins or any other members of the SLC4 family. In some structures of AE1 and the AE2(TMD)-AE3(CTD) chimera, CHOL molecules or phospholipids have been assigned in these two PIP_2_ binding areas (64, 67, 69). Other CHOL molecules have also been resolved in structures of AE1 and the AE2(TMD)-AE3(CTD) chimera. The number of bound CHOL molecules (1, 2, or 4), as well as their position on the TMD surface varies depending on the structure (76). CHOL molecules are found also at the interface between glycophorin A (GA) and the AE1 TMD in several AE1-GA complexes and AE1-ankyrin composites (65). Phospholipids have been resolved in multiple AE1 structures at the extracellular side of the dimeric interface formed by the extracellular portion of TM6 (64, 65, 67, 68).

Coarse-grained and atomistic MD simulations, which outline protein-lipid interactions and specific lipid binding sites, have been done for human AE1 (OF state) (68, 73, 74, 76, 118, 119), human AE1 (IF state) (68), and bovine AE1 (IF state), NBCe1 (OF state), and NDCBE (OF state) (76) with simplified model membranes representing erythrocyte or HEK293 membranes. AE1 simulations in phase-separated membranes indicate that AE1 segregates to a different extent in low and high-order lipid domains (75). The simulations demonstrate enrichment of PIP_2_ and PS lipids around the TMD of all studied SLC4 proteins, which form a negatively charged annulus at the extracellular side of the TMD with multiple long-lived PIP_2_ or PS coordination areas (73, 74, 76). Coarse-grained MD simulations of GA and AE1 containing membranes lead to the formation of GA/AE1 chains (73). The PIP_2_ binding site resolved in the cryoEM structures of AE1 and AE2 is also identified as site S1c_PIP2_ in the computational modeling studies in all studied proteins, including the Na^+^-dependent members of the family NBCe1 and NDCBE (Fig. 5) (76). In AE1, the PIP_2_ binding occurs both in the OF and IF state and in the same binding area (65, 67, 76). The PIP_2_ inositol headgroup is tucked under the cytoplasmic end of TMs 6 and 7 and stabilized by multiple positively charged residues (Fig. 5). Polar residues, such as Tyr, Gln, and Thr, which contribute further to the PIP_2_ headgroup coordination and a Pro residue which provides structural flexibility in the PIP_2_ binding site necessary for accommodating the PIP_2_ molecule, are also frequently found in this area. The PIP_2_ tails are coordinated by hydrophobic residues of TM7 and the loop between H4_TM_ and H5_TM_ but are not buried within the protein matrix as seen in multiple functionally relevant PIP_2_ sites in other proteins. The peripheral, solvent exposed PIP_2_ binding site resembles the one found in some inwardly rectifying K^+^ channels (Kir channels) (76, 120). Based on this comparison and the abundance of PIP_2_-bound SLC4 structures, it has been suggested that site S1c_PIP2_ is an actual functionally relevant binding site (76). Computational studies and PIP_2_ assays show that binding of PIP_2_ at this site increases the barrier of the OF↔IF transition in AE1, which inhibits AE1-mediated transport (68). The PIP_2_ headgroup is involved in interactions with positively charged residues of IL5 in the OF state which are lost in the IF state of AE1 when IL5 moves downward together with TM10 (68, 71). In addition, the S1c_PIP2_ binding site is found in the area of an anion reservoir identified in several SLC4 proteins (76). Thus, a possible explanation for the potential regulatory effect of PIP_2_ bound at the S1c_PIP2_ site includes modulation of anion entry into the IF cavity and of the OF↔IF conformational change.

Multiple CHOL binding sites were found in crevices between TMs 1, 2, 5, 6, 7, 9, 11, 13, H1_TM_, H3_TM_, and H4_TM_ of the TMD, including a long-lived CHOL site at TM1 and H3_TM_ in the IF bovine AE1 system, which is lodged between the gate and the core and might represent a previously proposed inhibitory CHOL site, which likely inhibits the IF→OF transition by locking the TMD in the IF state (the state in which AE1 is predominantly found in the erythrocyte membrane) (76). CHOL molecules are also frequently found at the cavity formed at the dimerization interface lined by TMs 5 and 6 (73, 74, 76). PMF calculations demonstrated that varying amounts of CHOL in model dipalmitoyl-phosphatidylcholine/dilinoleyl-phosphatidylcholine/CHOL membranes impact the energy of binding of the bicarbonate ion in site S1 due to changes in compression of the OF cavity (119). A recurrent binding site for phospholipids such as phosphatidylcholine and sphingomyelin is identified in AE1, NBCe1, and NDCBE at the same position as the phospholipid site identified in cryoEM structures of AE1 indicating that these lipids might be involved in SLC4 dimerization (76). Lipid–protein interactions have not been assessed computationally for any nonmammalian SLC4 transporters, although there are some indications of potential lipid role in dimerization of Bor1p (121). Considering the sparse functional and computational data on most mammalian SLC4 transporters and their plant and fungal borate analogs, the role of CHOL, PIP_2_, and phospholipids in the SLC4 transport mechanism (if such a role exists) remains poorly understood.

## Role of CTD in protein–protein and protein–lipid interactions, pH sensing, and conformational changes of mammalian SLC4

Early attempts at homology modeling of the full AE1 structure, provided models with different CTD orientation with respect to the TMD (74, 122). Considering the absence of CTD in most resolved mammalian SLC4 structures, it has been suggested that this is a highly flexible region, resulting from the long flexible linker between the TMD and CTD (Fig. 2) (63, 71). TMD and CTD so far have been resolved together in eleven OF human AE1 structures, two bovine (IF-IF and mixed OF-IF dimers) AE1 structures, three AE2 IF structures, a chimera IF AE2(TMD)-AE3(CTD) structure, and six BTR1 (OF and IF) structures (Table 1). The AE1 CTDs appear as dimers and show the same dimerization interface regardless of the originating TMD + CTD structure (Fig. 1*B*). This interface is also seen in older X-ray structures of the isolated CTD, which indicates that the CTD dimers are a functional unit, and their monomers do not move independently from one another (58, 59). A pH-dependent conformational equilibrium between a compact (at pH < 7.0) and an extended (at pH > 7.0) form of the AE1 CTD have been proposed based on a variety of methods (59); however, all reported X-ray and cryoEM AE1 CTD structures represent the compact form and the proposed structural features (short intramonomer distances between C201 and C317 and long intermonomer distances between W105 and D316) of the extended form cannot be verified.

**Figure 6:**
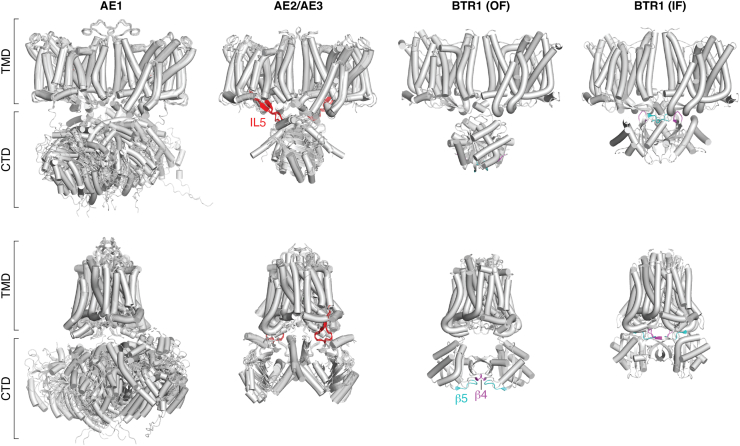
**Overlap of all available SLC4 structures where TMD and CTD were simultaneously resolved.** The figure illustrates the variability in the mutual orientation of CTD and TMD. The SLC4 structures are presented as *white cylinders*. IL5 in the AE2 structures, which has been suggested to anchor the CTD to the TMD in the IF AE2 states, is highlighted in *red*. In BTR1, the portions of beta sheets β4 and β5, which are proposed to lock the CTD to the TMD in the IF state, are highlighted in *magenta* and *cyan*, respectively. AE2, anion exchanger 2; BTR1, a human SLC4 transporter, corresponding to SLC4A11; CTD, cytoplasmic domain; IF, inward-facing state; IL5, intracellular loop 5; TMD, transmembrane domain; AE1, anion exchanger 1; AE3, anion exchanger 3.

The AE1 CTD dimers can assume multiple different positions in space with respect to the AE1 TMD dimers, depending on the presence of other intracellular proteins (Fig. 6). For instance, direct binding of the AE1 CTD to ankyrin repeats AR17 to 19 and 22 to 24 occurs when the CTD are in a V conformation with respect to the TMD (Fig. 1*B*), while the structures where AE1 is bound to intracellular protein 4.2, or resolved without bound proteins, favor a rev-V conformation with respect to the TMD (65, 66). A bovine IF AE1 structure with V conformation of the CTD with respect to the TMD was also resolved in absence of intracellular proteins (71); however, considering the overall resolution (6.30 Å) of this structure, it is possible that the CTDs were fitted inaccurately into the V conformation, instead of rev-V. Within the 10 human OF AE1 structures with rev-V conformation of the CTD, the CTDs adopt various tilted orientations with respect to the TMD, appear disengaged from the TMD, and can shift to the left or right boundaries of the TMD dimers (Fig. 6). Moreover, the rev-V CTD conformation is achieved by a ∼180° flip of the V CTD conformation with respect to the TMD, which indicates that the flexible linkers binding the CTD to the TMD are long enough to allow for such drastically different CTD orientations. This could explain the difficulty in the structural resolution of the CTD domains by cryoEM or X-ray in AE1 structures where intracellular proteins that can arrest the CTD in a specific and more easily resolved orientation are missing. Considering the absence of fully resolved structures of the Na^+^-dependent transporters of the family (NBCn1, NBCe1, and NDCBE were resolved as a TMD only, Table 1), it is likely that the flexibility of the flexible linkers that allows substantial motion to the CTD in the absence of intracellular proteins applies to the whole SLC4 family. In addition, a similar ∼180^o^ flip as seen in AE1 was also observed in BTR1 (43, 70), even though the BTR1 CTDs are substantially different in terms of size, structure, and sequence identity.

Interestingly, the existing three IF TMD + CTD AE2 structures (17, 18) and the chimera IF AE2(TMD)—AE3(CTD) structure (69) feature the CTD in close contact with the TMD, in a rev-V conformation and perpendicular with respect to the TMD dimer (Fig. 6). The CTD interacts with the TMD *via* IL5, which seemingly acts as an anchor locking the TMD in space (17). The IL5 can reach and interact with the CTD only if TM10 moves downward into a position consistent with the IF state. In the AE2 case, the interlocked CTD and TMD are observed over a pH range of 6.55 to 7.20. An alkaline pH (∼8.32) leads to decoupling of the CTD from the TMD and to resolution of IF AE2 dimers missing their CTD. This was suggested as evidence for a possible role of the AE2 CTDs as intracellular pH-sensors, leading to pH-dependent CTD/TMD reorganization, which can prepare the IF AE2 state to bind a HCO_3_^−^ ion and switch to an OF state (17). However, resolution of an IF AE2 structure without the CTD domains at physiological pH shows that this TMD-IL5-CTD interlocking is not a prerequisite of the formation of an IF state in AE2 and that CTD can be flexible and unresolvable at nonbasic pH as well (18). IF states were resolved without the CTD also in bovine and human AE1 (68, 71) and in IF BTR1 the IL5-CTD contact is not evident (43).

The role of the CTD in the SLC4 function is still unclear. Substrate transport is retained in truncated AE1, with absent CTD (3, 123). The plant and yeast Bor1 transporters do not have a CTD like the one found in the mammalian SLC4 members (45, 47, 48). Kidney AE1 differs from erythrocyte AE1 in that it is missing residues 1 to 65, which include the N_t_ tail and part of the CTD (124). This truncation impacts its ability to bind ankyrin, protein 4.1, or glycolytic enzymes but does not appear to impact its anion exchange function (TOR values for both kidney and erythrocyte AE1 are > 60,000 s^−1^) (102). Other than the obvious interactions with the intracellular proteins (which have been mostly studied in erythrocyte AE1) and the presumed pH sensing role of the CTD in AE2, there are indications of functional impact of the CTD on the TMD-mediated anion transport (*e.g.*, the Memphis phenotype mutation K56E in human AE1 CTD leads to 20% decrease in phosphoenolpyruvate transport by the TMD (125, 126)). Early studies in AE1 have proposed the existence of a substrate tunnel through the CTD from which the anions can reach the TMD (59). Such a tunnel was not resolved in any of the CTD structures available today and does not appear necessary, considering the observed flexibility of the CTD. Ion permeation calculations with full SLC4 structures have not been published, but the previously established permeation pathways in IF AE1 and NBCn1 do not appear to be shielded by the available positions of the CTD with respect to the TMD (62, 71). In BTR1, structures and cryoEM maps with CTD and TMD were resolved in both the OF and IF state in WT BTR1 at different pH (43, 70). The OF BTR1 features a PIP_2_ molecule bound at the interface between the CTD and TM13, H4_TM_, and H5_TM_ from the TMD. An R125H mutation in the CTD or acidic conditions (pH ∼5.5) provide structures in the IF state in which the PIP_2_ molecule is missing, and the CTD has undergone a ∼180^o^ flip during which the β4 and β5 sheets (Fig. 6) start interacting with the TMD gate domain, presumably stabilizing it in the IF state. As a result, a role of the CTD in pH-mediated PIP_2_ binding and stabilization of the IF state has been suggested. In AE2, however, presence of PIP_2_ presumably unanchors the CTD from the TMD, and the CTD cannot be resolved (18). In the absence of more detailed functional and computational studies, it is not yet clear if the observed interactions between the IL5 and CTD in AE2 or β4 and β5 and the gate domain in BTR1 can block or modulate the OF↔IF transition, and if any of these interactions can be translated to other members of the SLC4 family. Thus, the structural and functional evidence on the interplay between the TMD/CTD organization, pH sensing, PIP_2_ binding, and modulation of the OF↔IF transition in SLC4 remains inconclusive.

## Conclusions and outlook

Rapid advances in cryoEM and computational methods in the last decade led to the resolution of various SLC4 structures, in the OF, IF, and occluded conformation and in the apo-state or states bound to substrate ions, inhibitors, cytoplasmic proteins, and/or lipids. This prompted computational studies of the complex biochemical and biophysical processes underpinning ion permeation, ion binding, and conformational changes, necessary for SLC4 substrate translocation through the membrane. In this review, we presented the major findings on the structure-function relationships in the SLC4 family obtained from a myriad of recently published SLC4 structures and computational studies. The most important accomplishments in the SLC4 field have been summarized below, and questions that need further elucidation have been highlighted.

i) The overall structure of the SLC4 transporters is now well established. The SLC4 proteins are dimers in which the TMD, that can be divided into a gate and a core domain, is involved in direct ion transport through the cellular membrane, while the CTD is involved in interactions with intracellular proteins and, aided by the long flexible linker, can assume a multitude of different orientations with respect to the TMD depending on the presence of intracellular proteins, membrane lipids, conformational state of the TMD, or intracellular pH. However, since all structural studies that produced SLC4 complexes with intracellular proteins were done with erythrocyte AE1, there is virtually no structural data for intracellular protein complexes of any of the other members of the family, including other AE1 isoforms, such as the kidney AE1 which has marked differences in its interactions with intracellular proteins when compared to erythrocyte AE1. In addition, the evidence of pH and lipid modulation of the TMD/CTD association/dissociation comes from a very small number of cryoEM structures and limited experimental data, and the conclusions drawn from these studies are yet to be categorically proven for the proteins for which these structures were resolved, let alone for the overall SLC4 family. Moreover, some of the conclusions (*e.g.*, PIP_2_ effect on CTD/TMD association in AE2 and BTR1) are contradictory, and in the absence of PIP_2_ transport assays or appropriate modeling studies, it is impossible to assess if this contradiction is circumstantial or a result of actual functional differences within the SLC4 family. The regulatory role of the CTD on the TMD function also remains poorly understood, and although the multitude of new structures, which include both TMD and CTD for several members of the family, is a very positive development in this area of research, much more experimental and modeling work would be required to understand how the CTD impact TMD transport and why single point mutations in CTD have a profound effect on SLC4 activity while at the same time there is evidence that the TMD retains function in truncated proteins lacking the CTD or in the borate transporters of the family, where the CTD is missing. In addition, TMD + CTD structures for the Na^+^-dependent members of the family do not exist yet, and it is not clear how much the CTD of these members of the family are structurally similar to the ones resolved for the anion exchangers or BTR1 and whether the same V and rev-V conformations are possible within them. Multiple questions also remain about the roles of N_t_ and C_t_ in the SLC4 regulation and self-inhibition, considering the flexibility of these regions which makes them difficult for both cryoEM and modeling assessment. More functional, structural, and computational work must also be done for identification of putative binding sites for different modulators of SLC4 activity which may bind to N_t_ and C_t_.
ii) The elevator transport in the SLC4 family is now well established and consistently demonstrated with OF and IF structures in several different members of the family. Small vertical translocation (∼5 Å) of the central binding residues and the rigid motion of the core with respect to the gate domain, aided by H2_TM_ and H3_TM_, likely leads to small protein and lipid reorganization with small energetic penalties, which can explain the efficient and remarkably fast transport (turnover rates of the order of 10^4^ s^−1^) observed in members of the SLC4 family. However, other than the OF and IF structures and limited modeling of the OF↔IF conformational transition, there is very little available information on the energetics and kinetics of transport in the SLC4 family and no information about the membrane reorganization effects on this transport. There are no identified SLC4 intermediate states other than an intermediate occluded state from modeling on NBCn1 and a few putative occluded cryoEM and X-ray structures of AtBor1. No true intermediate experimental structures have yet been determined for the Na⁺-dependent and -independent mammalian SLC4 transporters. The structural and modeling evidence suggests that the occlusion in the OF, IF, and intermediate states occurs from alternating interactions between hydrophobic residues forming hydrophobic contact interfaces in the vicinity of the central binding site. However, it is not clear if there are other, more specific gating events and local reorganizations in these interfaces that would be required for occlusion and translocation. The IF AE1 states with unfolded TM10 and elongated TM11 (called here “inactivated states” as a suggestion and without supporting evidence) and the self-inhibited AE2 and AtBor1 states (also named “self-inhibited states” without supporting evidence) are yet to be put in the context of the full transport cycle and assessment of their role requires further work. The changes in the TM10 and 11 in the “inactivated state” and occluded AtBor1 would be expected to be slow, and this contradicts the observed high transport rates in the SLC4 family. Most importantly, there are no available cryoEM structures for the IF states of the Na^+^-dependent members of the family, and it is not clear if homology models based on the IF Na^+^-independent exchangers can fully capture the features of the IF Na^+^-dependent SLC4 states.
iii) Substrate binding sites have now been resolved from structural and computational studies for several members of the mammalian SLC4 family, both Na^+^-dependent symporters and exchangers and Na^+^-independent exchangers. The central binding sites in the Na^+^-independent exchangers of the family have been resolved both in the OF and IF states and provide direct evidence for the rigid translocation of the site with little protein reorganization during the OF ↔ IF translocation. Whether the same structural details apply to Na⁺-dependent mammalian SLC4 transporters remains unresolved because experimentally determined IF structures are not yet available for these members. Confirmation of computationally predicted SLC4 central binding sites by cryoEM structures was also done, which demonstrates the impressive predictive capabilities of the current modeling methods and raises the confidence in the computationally identified additional putative entry ion/substrate binding sites in the OF and IF states of some Na^+^-dependent and -independent transporters of the family, for which there is no structural evidence yet. Nevertheless, the role of the entry and central sites in substrate selectivity, thermodynamics of binding, kinetics, and thermodynamics of permeation, and pH-dependent transport is still unclear and requires considerable further experimental and computational work. In the Na^+^-dependent transporters, the order of ion binding and its relevance to transport is yet to be established. The local and global effects upon ion binding in the entry and central sites and how the OF ↔ IF transition is triggered are not clear. In the absence of IF states for the Na^+^-dependent members of the family and as a result, absence of structurally and functionally confirmed IF binding sites, even questions such as transport stoichiometry cannot yet be answered in some cases (*e.g.*, it is not clear how the experimentally proposed Na^+^ + CO_3_^2^^−^ + HCO_3_^−^ ion combination binds and is transported in NBCe1 and under what structural and environmental *in vivo* circumstances). The BTR1 member of the family remains an enigma and even the OF and IF structures resolved for it could not fully explain its function. Computational modeling with advanced computational methods and extensive functional investigation would be required for studies of the proposed NH_4_^+^, NH_3_, H^+^, and OH^−^ transport by BTR1 and its role in health and disease. The borate transporters of the family are especially understudied, and there are no computationally identified binding sites and permeation pathways for borate and protons in AtBor1 or Bor1p, despite the available occluded and IF structures.

Generally, very little is known about the structure-function effect of known disease-causing mutations in the mammalian SLC4 family both due to absence of structures of SLC4 mutants and insufficient functional studies. Binding sites for a number of nonspecific SLC4 inhibitors were resolved from cryoEM. Improved understanding of inhibitor binding may aid in the development of member- and state-specific inhibitors and activators of the SLC4 members implicated in human diseases, and this promising avenue of SLC4 research is only just beginning. Another promising avenue of SLC4 research for pharmacological applications, which is in its infancy, is the lipid modulation of SLC4 transport. Despite the resolution of binding sites for membrane components such as phospholipids, CHOL, and PIP_2_-like lipids in multiple SLC4 proteins, there is very little functional and computational evidence for lipid effect on SLC4 function. Where such experimental evidence exists, such as the CHOL effect on AE1 and PIP_2_ effect on NBCe1, the information about the structural underpinnings of these effects is sorely lacking. Recent promising trends in investigations of membrane-modulated protein function in other membrane proteins, such as potassium ion channels, include effect of polyunsaturated fatty acids, endocannabinoids, or membrane soluble pharmaceuticals and highlight an untapped potential for similar studies in the SLC4 transporters, where these effects have not been investigated yet.

Overall, despite the major developments in the structure-function relationships in the SLC4 family, knowledge is still lacking in some critical areas. CryoEM emerges as one of the most important and useful tools for structural studies, and considering that the majority of the SLC4 structures reviewed here were published in the last 5 years, we expect to see many more SLC4 structures in the near future. On the computational front, the increasing computational power of the new processors and graphic cards, the improved modeling and analytical software, and the new machine learning methods and tools are expected to deliver unprecedented detail on the structural underpinnings of protein function. We hope that the present review both illustrates the immense potential of combined structural, functional, and computational studies for elucidation of SLC4 function and provides useful guidance for future endeavors in this direction.

## Conflict of interest

The authors declare that they have no conflicts of interest with the contents of this article.

## References

[1] Kurtz I. (2014). NBCe1 as a model carrier for understanding the structure-function properties of Na^+^-coupled SLC4 transporters in health and disease. Pflugers Arch..

[2] Parker M.D., Boron W.F. (2013). The divergence, actions, roles, and relatives of sodium-coupled bicarbonate transporters. Physiol. Rev..

[3] Reithmeier R.A., Casey J.R., Kalli A.C., Sansom M.S., Alguel Y., Iwata S. (2016). Band 3, the human red cell chloride/bicarbonate anion exchanger (AE1, SLC4A1), in a structural context. Biochim. Biophys. Acta.

[4] Romero M.F., Chen A.P., Parker M.D., Boron W.F. (2013). The SLC4 family of bicarbonate HCO_3_^−^ transporters. Mol. Aspects Med..

[5] Beltran J.L., McGrath L.G., Caruso S., Bain R.K., Hendrix C.E., Kamran H. (2023). Borate transporters and SLC4 bicarbonate transporters share key functional properties. Membranes (Basel).

[6] Alka K., Casey J.R. (2014). Bicarbonate transport in health and disease. IUBMB Life.

[7] Alper S.L. (2009). Molecular physiology and genetics of Na^+^-independent SLC4 anion exchangers. J. Exp. Biol..

[8] Choi I., Aalkjaer C., Boulpaep E.L., Boron W.F. (2000). An electroneutral sodium/bicarbonate cotransporter NBCn1 and associated sodium channel. Nature.

[9] Liu Y., Yang J., Chen L.M. (2015). Structure and function of SLC4 family HCO_3_ transporters. Front. Physiol..

[10] Pushkin A., Kurtz I. (2006). SLC4 base (HCO_3_^−^, CO_3_^2−^) transporters: classification, function, structure, genetic diseases, and knockout models. Am. J. Physiol. Ren. Physiol..

[11] Pena-Munzenmayer G., George A.T., Shull G.E., Melvin J.E., Catalan M.A. (2016). Ae4 (Slc4a9) is an electroneutral monovalent cation-dependent Cl^−^/HCO_3_^−^ exchanger. J. Gen. Physiol..

[12] Kao L., Azimov R., Shao X.M., Abuladze N., Newman D., Zhekova H. (2020). SLC4A11 function: evidence for H^+^ OH^−^ and NH_3_^−^ H^+^ transport. Am. J. Physiol. Cell Physiol..

[13] Kovaleva P.A., Kotova E.S., Sharova E.I., Skorodumova L.O. (2025). SLC4A11 revisited: isoforms, expression, functions, and unresolved questions. Biomolecules.

[14] Kurtz I., Schwartz G.J. (2023). Base (HCO_3_^−^/CO_3_^2−^) transport properties of SLC4 proteins: new insights in acid-base kidney physiology. J. Am. Soc. Nephrol..

[15] Parker M.D. (2018). Mouse models of SLC4-linked disorders of HCO_3_^−^-transporter dysfunction. Am. J. Physiol. Cell Physiol..

[16] Medina J.F. (2011). Role of the anion exchanger 2 in the pathogenesis and treatment of primary biliary cirrhosis. Dig. Dis..

[17] Zhang Q., Jian L., Yao D., Rao B., Xia Y., Hu K. (2023). The structural basis of the pH-homeostasis mediated by the Cl^−^/HCO_3_^−^ exchanger, AE2. Nat. Commun..

[18] Zhang W., Ding D., Lu Y., Chen H., Jiang P., Zuo P. (2024). Structural and functional insights into the lipid regulation of human anion exchanger 2. Nat. Commun..

[19] Casey J.R., Sly W.S., Shah G.N., Alvarez B.V. (2009). Bicarbonate homeostasis in excitable tissues: role of AE3 Cl^−^/HCO_3_^−^ exchanger and carbonic anhydrase XIV interaction. Am. J. Physiol. Cell Physiol..

[20] Christiansen M.K., Kjaer-Sorensen K., Clavsen N.C., Dittmann S., Jensen M.F., Guldbrandsen H.O. (2023). Genetic analysis identifies the SLC4A3 anion exchanger as a major gene for short QT syndrome. Heart Rhythm.

[21] Hentschke M., Wiemann M., Hentschke S., Kurth I., Hermans-Borgmeyer I., Seidenbecher T. (2006). Mice with a targeted disruption of the Cl^−^/HCO_3_^−^ exchanger AE3 display a reduced seizure threshold. Mol. Cell Biol..

[22] Sowah D., Brown B.F., Quon A., Alvarez B.V., Casey J.R. (2014). Resistance to cardiomyocyte hypertrophy in ae3-/- mice, deficient in the AE3 Cl^−^/HCO_3_^−^ exchanger. BMC Cardiovasc. Disord..

[23] Su Y.R., Klanke C.A., Houseal T.W., Linn S.C., Burk S.E., Varvil T.S. (1994). Molecular cloning and physical and genetic mapping of the human anion exchanger isoform 3 (SLC2C) gene to chromosome 2q36. Genomics.

[24] Thorsen K., Dam V.S., Kjaer-Sorensen K., Pedersen L.N., Skeberdis V.A., Jurevicius J. (2017). Loss-of-activity-mutation in the cardiac chloride-bicarbonate exchanger AE3 causes short QT syndrome. Nat. Commun..

[25] Vilas G.L., Johnson D.E., Freund P., Casey J.R. (2009). Characterization of an epilepsy-associated variant of the human Cl^−^/HCO_3_^−^ exchanger AE3. Am. J. Physiol. Cell Physiol..

[26] Brady C.T., Marshall A., Zhang C., Parker M.D. (2023). NBCe1-B/C-knockout mice exhibit an impaired respiratory response and an enhanced renal response to metabolic acidosis. Front. Physiol..

[27] Saint-Criq V., Guequen A., Philp A.R., Villanueva S., Apablaza T., Fernandez-Moncada I. (2022). Inhibition of the sodium-dependent HCO_3_^−^ transporter SLC4A4, produces a cystic fibrosis-like airway disease phenotype. Elife.

[28] Theparambil S.M., Ruminot I., Schneider H.P., Shull G.E., Deitmer J.W. (2014). The electrogenic sodium bicarbonate cotransporter NBCe1 is a high-affinity bicarbonate carrier in cortical astrocytes. J. Neurosci..

[29] Barbuskaite D., Pedersen F.D., Christensen H.L., Johnsen L.O., Praetorius J., Damkier H.H. (2020). NBCe2 (Slc4a5) is expressed in the renal connecting tubules and cortical collecting ducts and mediates base extrusion. Front. Physiol..

[30] Christensen H.L., Barbuskaite D., Rojek A., Malte H., Christensen I.B., Fuchtbauer A.C. (2018). The choroid plexus sodium-bicarbonate cotransporter NBCe2 regulates mouse cerebrospinal fluid pH. J. Physiol..

[31] Gildea J.J., Xu P., Kemp B.A., Carlson J.M., Tran H.T., Bigler Wang D. (2018). Sodium bicarbonate cotransporter NBCe2 gene variants increase sodium and bicarbonate transport in human renal proximal tubule cells. PLoS One.

[32] Kao L., Kurtz L.M., Shao X., Papadopoulos M.C., Liu L., Bok D. (2011). Severe neurologic impairment in mice with targeted disruption of the electrogenic sodium bicarbonate cotransporter NBCe2 (Slc4a5 gene). J. Biol. Chem..

[33] Peng T., Li B., Bi L., Zhang F. (2024). Iguratimod inhibits protein citrullination and inflammation by downregulating NBCe2 in patients with rheumatoid arthritis. Biomed. Pharmacother..

[34] Axelsen T.V., Olesen C., Khan D., Mohammadi A., Bouzinova E.V., Nielsen C.J.F. (2024). Antibodies toward Na^+^, HCO_3_^−^-cotransporter NBCn1/SLC4A7 block net acid extrusion and cause pH-dependent growth inhibition and apoptosis in breast cancer. Br. J. Cancer.

[35] Boedtkjer E., Praetorius J., Aalkjaer C. (2006). NBCn1 (slc4a7) mediates the Na^+^-dependent bicarbonate transport important for regulation of intracellular pH in mouse vascular smooth muscle cells. Circ. Res..

[36] Lee H.J., Kwon M.H., Lee S., Hall R.A., Yun C.C., Choi I. (2014). Systematic family-wide analysis of sodium bicarbonate cotransporter NBCn1/SLC4A7 interactions with PDZ scaffold proteins. Physiol. Rep..

[37] Ng F.L., Boedtkjer E., Witkowska K., Ren M., Zhang R., Tucker A. (2017). Increased NBCn1 expression, Na^+^/HCO_3_^−^ co-transport and intracellular pH in human vascular smooth muscle cells with a risk allele for hypertension. Hum. Mol. Genet..

[38] Olesen C.W., Vogensen J., Axholm I., Severin M., Schnipper J., Pedersen I.S. (2018). Trafficking, localization and degradation of the Na^+^, HCO_3_^−^ co-transporter NBCn1 in kidney and breast epithelial cells. Sci. Rep..

[39] Wang J., Zahra A., Wang Y., Wu J. (2022). Understanding the physiological role of electroneutral Na^+^-coupled HCO_3_^−^ cotransporter and its therapeutic implications. Pharmaceuticals (Basel).

[40] Lee H.J., Park H.J., Lee S., Kim Y.H., Choi I. (2011). The sodium-driven chloride/bicarbonate exchanger NDCBE in rat brain is upregulated by chronic metabolic acidosis. Brain Res..

[41] Vitzthum H., Koch M., Eckermann L., Svendsen S.L., Berg P., Hubner C.A. (2023). The AE4 transporter mediates kidney acid-base sensing. Nat. Commun..

[42] Parker M.D., Musa-Aziz R., Rojas J.D., Choi I., Daly C.M., Boron W.F. (2008). Characterization of human SLC4A10 as an electroneutral Na/HCO3 cotransporter (NBCn2) with Cl^−^ self-exchange activity. J. Biol. Chem..

[43] Lu Y., Zuo P., Chen H., Shan H., Wang W., Dai Z. (2023). Structural insights into the conformational changes of BTR1/SLC4A11 in complex with PIP(2). Nat. Commun..

[44] Patel S.P., Parker M.D. (2015). SLC4A11 and the pathophysiology of congenital hereditary endothelial dystrophy. Biomed. Res. Int..

[45] Thurtle-Schmidt B.H., Stroud R.M. (2016). Structure of Bor1 supports an elevator transport mechanism for SLC4 anion exchangers. Proc. Natl. Acad. Sci. U.S.A..

[46] Tanaka M., Fujiwara T. (2008). Physiological roles and transport mechanisms of boron: perspectives from plants. Pflugers Arch..

[47] Saouros S., Mohan T.C., Cecchetti C., Lehmann S., Barrit J.D., Scull N.J. (2021). Structural and functional insights into the mechanism of action of plant borate transporters. Sci. Rep..

[48] Jiang Y., Jiang J. (2024). The Bor1 elevator transport cycle is subject to autoinhibition and activation. Nat. Commun..

[49] Coudray N., S L.S., Lasala R., Zhang Z., Clark K.M., Dumont M.E. (2017). Structure of the SLC4 transporter Bor1p in an inward-facing conformation. Protein Sci..

[50] Dolder M., Walz T., Hefti A., Engel A. (1993). Human erythrocyte band 3. Solubilization and reconstitution into two-dimensional crystals. J. Mol. Biol..

[51] Wang D.N., Kuhlbrandt W., Sarabia V.E., Reithmeier R.A. (1993). Two-dimensional structure of the membrane domain of human band 3, the anion transport protein of the erythrocyte membrane. EMBO J..

[52] Wang D.N., Sarabia V.E., Reithmeier R.A., Kuhlbrandt W. (1994). Three-dimensional map of the dimeric membrane domain of the human erythrocyte anion exchanger, Band 3. EMBO J..

[53] Gargaro A.R., Bloomberg G.B., Dempsey C.E., Murray M., Tanner M.J. (1994). The solution structures of the first and second transmembrane-spanning segments of band 3. Eur. J. Biochem..

[54] Schneider M.L., Post C.B. (1995). Solution structure of a band 3 peptide inhibitor bound to aldolase: a proposed mechanism for regulating binding by tyrosine phosphorylation. Biochemistry.

[55] Eisenmesser E.Z., Post C.B. (1998). Insights into tyrosine phosphorylation control of protein-protein association from the NMR structure of a band 3 peptide inhibitor bound to glyceraldehyde-3-phosphate dehydrogenase. Biochemistry.

[56] Askin D., Bloomberg G.B., Chambers E.J., Tanner M.J. (1998). NMR solution structure of a cytoplasmic surface loop of the human red cell anion transporter, band 3. Biochemistry.

[57] Chambers E.J., Bloomberg G.B., Ring S.M., Tanner M.J. (1999). Structural studies on the effects of the deletion in the red cell anion exchanger (band 3, AE1) associated with South East Asian ovalocytosis. J. Mol. Biol..

[58] Zhang D., Kiyatkin A., Bolin J.T., Low P.S. (2000). Crystallographic structure and functional interpretation of the cytoplasmic domain of erythrocyte membrane band 3. Blood.

[59] Shnitsar V., Li J., Li X., Calmettes C., Basu A., Casey J.R. (2013). A substrate access tunnel in the cytosolic domain is not an essential feature of the solute carrier 4 (SLC4) family of bicarbonate transporters. J. Biol. Chem..

[60] Arakawa T., Kobayashi-Yurugi T., Alguel Y., Iwanari H., Hatae H., Iwata M. (2015). Crystal structure of the anion exchanger domain of human erythrocyte band 3. Science.

[61] Huynh K.W., Jiang J., Abuladze N., Tsirulnikov K., Kao L., Shao X. (2018). CryoEM structure of the human SLC4A4 sodium-coupled acid-base transporter NBCe1. Nat. Commun..

[62] Wang W., Zhekova H.R., Tsirulnikov K., Dwadasi B.S., Aparicio E.G., Azimov R. (2025). CryoEM and computational modelling structural insights into the pH regulator NBCn1. Nat. Commun..

[63] Wang W., Tsirulnikov K., Zhekova H.R., Kayik G., Khan H.M., Azimov R. (2021). Cryo-EM structure of the sodium-driven chloride/bicarbonate exchanger NDCBE. Nat. Commun..

[64] Capper M.J., Yang S., Stone A.C., Vatansever S., Zilberg G., Mathiharan Y.K. (2023). Substrate binding and inhibition of the anion exchanger 1 transporter. Nat. Struct. Mol. Biol..

[65] Vallese F., Kim K., Yen L.Y., Johnston J.D., Noble A.J., Cali T. (2022). Architecture of the human erythrocyte ankyrin-1 complex. Nat. Struct. Mol. Biol..

[66] Xia X., Liu S., Zhou Z.H. (2022). Structure, dynamics and assembly of the ankyrin complex on human red blood cell membrane. Nat. Struct. Mol. Biol..

[67] Su C.C., Zhang Z., Lyu M., Cui M., Yu E.W. (2024). Cryo-EM structures of the human band 3 transporter indicate a transport mechanism involving the coupled movement of chloride and bicarbonate ions. PLoS Biol..

[68] Chen T., Vallese F., Gil-Iturbe E., Kim K., Cali T., Quick M. (2025). Impact of anionic lipids on the energy landscape of conformational transition in anion exchanger 1 (AE1). Nat. Commun..

[69] Jian L., Zhang Q., Yao D., Wang Q., Chen M., Xia Y. (2024). The structural insight into the functional modulation of human anion exchanger 3. Nat. Commun..

[70] Liu C., Chen X., Chang L., Li T., Hao Z., Wang Z. (2025). Cryo-EM structures and transport mechanism of human multifunctional transporter BTR1. Protein Cell.

[71] Zhekova H.R., Jiang J., Wang W., Tsirulnikov K., Kayik G., Khan H.M. (2022). CryoEM structures of anion exchanger 1 capture multiple states of inward- and outward-facing conformations. Commun. Biol..

[72] Zhekova H.R., Pushkin A., Kayik G., Kao L., Azimov R., Abuladze N. (2021). Identification of multiple substrate binding sites in SLC4 transporters in the outward-facing conformation: insights into the transport mechanism. J. Biol. Chem..

[73] Kalli A.C., Reithmeier R.A.F. (2018). Interaction of the human erythrocyte Band 3 anion exchanger 1 (AE1, SLC4A1) with lipids and glycophorin A: molecular organization of the Wright (Wr) blood group antigen. PLoS Comput. Biol..

[74] De Vecchis D., Reithmeier R.A.F., Kalli A.C. (2019). Molecular simulations of intact anion exchanger 1 reveal specific domain and lipid interactions. Biophys. J..

[75] Jin Y., Liang Q., Tieleman D.P. (2020). Interactions between band 3 anion exchanger and lipid nanodomains in ternary lipid bilayers: atomistic simulations. J. Phys. Chem. B.

[76] Zhekova H.R., Ramirez Echemendia D.P., Sejdiu B.I., Pushkin A., Tieleman D.P., Kurtz I. (2024). Molecular dynamics simulations of lipid-protein interactions in SLC4 proteins. Biophys. J..

[77] Catalan M.A., Flores-Aldama L., Fernandez F., Bustos D., Apablaza N., Hidalgo A. (2025). Molecular determinants of [Formula: see text] and cation transport in the human cation-dependent Cl^−^/[Formula: see text] exchanger AE4. Am. J. Physiol. Cell Physiol..

[78] Vince J.W., Reithmeier R.A. (1998). Carbonic anhydrase II binds to the carboxyl terminus of human band 3, the erythrocyte Cl^−^/HCO_3_^−^ exchanger. J. Biol. Chem..

[79] Vince J.W., Reithmeier R.A. (2000). Identification of the carbonic anhydrase II binding site in the Cl^−^/HCO_3_^−^ anion exchanger AE1. Biochemistry.

[80] Hong J.H., Yang D., Shcheynikov N., Ohana E., Shin D.M., Muallem S. (2013). Convergence of IRBIT, phosphatidylinositol (4,5) bisphosphate, and WNK/SPAK kinases in regulation of the Na^+^-HCO_3_^−^ cotransporters family. Proc. Natl. Acad. Sci. U.S.A..

[81] Thornell I.M., Bevensee M.O. (2015). Regulators of Slc4 bicarbonate transporter activity. Front. Physiol..

[82] Hashimoto K., Panchenko A.R. (2010). Mechanisms of protein oligomerization, the critical role of insertions and deletions in maintaining different oligomeric states. Proc. Natl. Acad. Sci. U.S.A..

[83] Gabizon R., Friedler A. (2014). Allosteric modulation of protein oligomerization: an emerging approach to drug design. Front. Chem..

[84] Kim S., Brandon S., Zhou Z., Cobb C.E., Edwards S.J., Moth C.W. (2011). Determination of structural models of the complex between the cytoplasmic domain of erythrocyte band 3 and ankyrin-R repeats 13-24. J. Biol. Chem..

[85] Sergeev M., Godin A.G., Kao L., Abuladze N., Wiseman P.W., Kurtz I. (2012). Determination of membrane protein transporter oligomerization in native tissue using spatial fluorescence intensity fluctuation analysis. PLoS One.

[86] Kao L., Sassani P., Azimov R., Pushkin A., Abuladze N., Peti-Peterdi J. (2008). Oligomeric structure and minimal functional unit of the electrogenic sodium bicarbonate cotransporter NBCe1-A. J. Biol. Chem..

[87] Macara I.G., Cantley L.C. (1981). Interactions between transport inhibitors at the anion binding sites of the band 3 dimer. Biochemistry.

[88] Ruan Y., Miyagi A., Wang X., Chami M., Boudker O., Scheuring S. (2017). Direct visualization of glutamate transporter elevator mechanism by high-speed AFM. Proc. Natl. Acad. Sci. U.S.A..

[89] Chang Y.N., Geertsma E.R. (2017). The novel class of seven transmembrane segment inverted repeat carriers. Biol. Chem..

[90] Ficici E., Faraldo-Gomez J.D., Jennings M.L., Forrest L.R. (2017). Asymmetry of inverted-topology repeats in the AE1 anion exchanger suggests an elevator-like mechanism. J. Gen. Physiol..

[91] Drew D., Boudker O. (2016). Shared molecular mechanisms of membrane transporters. Annu. Rev. Biochem..

[92] Garaeva A.A., Slotboom D.J. (2020). Elevator-type mechanisms of membrane transport. Biochem. Soc. Trans..

[93] Parker M.D., Bouyer P., Daly C.M., Boron W.F. (2008). Cloning and characterization of novel human SLC4A8 gene products encoding Na+-driven Cl^−^/HCO_3_^−^ exchanger variants NDCBE-A, -C, and -D. Physiol. Genomics.

[94] Alguel Y., Amillis S., Leung J., Lambrinidis G., Capaldi S., Scull N.J. (2016). Structure of eukaryotic purine/H^+^ symporter UapA suggests a role for homodimerization in transport activity. Nat. Commun..

[95] Lu F., Li S., Jiang Y., Jiang J., Fan H., Lu G. (2011). Structure and mechanism of the uracil transporter UraA. Nature.

[96] Passow H. (1986). Molecular aspects of band 3 protein-mediated anion transport across the red blood cell membrane. Rev. Physiol. Biochem. Pharmacol..

[97] Tanford C. (1985). Simple model can explain self-inhibition of red cell anion exchange. Biophys. J..

[98] Wu H., Liu S., Su P., Xie Z.D., Gui T.X., Zhao L. (2022). Molecular insight into coordination sites for substrates and their coupling kinetics in Na^+^/HCO_3_^−^ cotransporter NBCe1. J. Physiol..

[99] Grimsley G.R., Scholtz J.M., Pace C.N. (2009). A summary of the measured pK values of the ionizable groups in folded proteins. Protein Sci..

[100] Jennings M.L., Smith J.S. (1992). Anion-proton cotransport through the human red blood cell band 3 protein. Role of glutamate 681. J. Biol. Chem..

[101] Chernova M.N., Jiang L., Crest M., Hand M., Vandorpe D.H., Strange K. (1997). Electrogenic sulfate/chloride exchange in Xenopus oocytes mediated by murine AE1 E699Q. J. Gen. Physiol..

[102] Pushkin A., Kao L., Zhekova H.R., Azimov R., Abuladze N., Shao X.M. (2024). On the substrate turnover rate of NBCe1 and AE1 SLC4 transporters: structure-function considerations. Front. Physiol..

[103] Yang H.S., Kim E., Lee S., Park H.J., Cooper D.S., Rajbhandari I. (2009). Mutation of aspartate 555 of the sodium/bicarbonate transporter SLC4A4/NBCe1 induces chloride transport. J. Biol. Chem..

[104] Lee S., Lin J., Choi I. (2022). Lack of charge interaction in the ion binding site determines anion selectivity in the sodium bicarbonate cotransporter NBCe1. Int. J. Mol. Sci..

[105] Jin X.R., Abe Y., Li C.Y., Hamasaki N. (2003). Histidine-834 of human erythrocyte band 3 has an essential role in the conformational changes that occur during the band 3-mediated anion exchange. Biochemistry.

[106] Izuhara K., Okubo K., Hamasaki N. (1989). Conformational change of band 3 protein induced by diethyl pyrocarbonate modification in human erythrocyte ghosts. Biochemistry.

[107] Falke J.J., Chan S.I. (1986). Molecular mechanisms of band 3 inhibitors. 2. Channel blockers. Biochemistry.

[108] Falke J.J., Chan S.I. (1986). Molecular mechanisms of band 3 inhibitors. 3. Translocation inhibitors. Biochemistry.

[109] Golan D.E., Alecio M.R., Veatch W.R., Rando R.R. (1984). Lateral mobility of phospholipid and cholesterol in the human erythrocyte membrane: effects of protein-lipid interactions. Biochemistry.

[110] Seigneuret M., Favre E., Morrot G., Devaux P.F. (1985). Strong interactions between a spin-labeled cholesterol analog and erythrocyte proteins in the human erythrocyte membrane. Biochim. Biophys. Acta.

[111] Schubert D., Boss K. (1982). Band 3 protein-cholesterol interactions in erythrocyte membranes. Possible role in anion transport and dependency on membrane phospholipid. FEBS Lett..

[112] Schubert D., Marie H. (1982). The structure of mixed cholesterol-phospholipid monolayers spread at the air-water interface as probed by interactions with band 3 protein from erythrocyte membranes. Biochim. Biophys. Acta.

[113] Pap E.H., Hanicak A., van Hoek A., Wirtz K.W., Visser A.J. (1995). Quantitative analysis of lipid-lipid and lipid-protein interactions in membranes by use of pyrene-labeled phosphoinositides. Biochemistry.

[114] Kohne W., Deuticke B., Haest C.W. (1983). Phospholipid dependence of the anion transport system of the human erythrocyte membrane. Studies on reconstituted band 3/lipid vesicles. Biochim. Biophys. Acta.

[115] Thornell I.M., Bevensee M.O. (2015). Phosphatidylinositol 4,5-bisphosphate degradation inhibits the Na^+^/bicarbonate cotransporter NBCe1-B and -C variants expressed in Xenopus oocytes. J. Physiol..

[116] Thornell I.M., Wu J., Liu X., Bevensee M.O. (2012). PIP2 hydrolysis stimulates the electrogenic Na^+^-bicarbonate cotransporter NBCe1-B and -C variants expressed in Xenopus laevis oocytes. J. Physiol..

[117] Wu J., McNicholas C.M., Bevensee M.O. (2009). Phosphatidylinositol 4,5-bisphosphate (PIP2) stimulates the electrogenic Na/HCO_3_ cotransporter NBCe1-A expressed in Xenopus oocytes. Proc. Natl. Acad. Sci. U.S.A..

[118] Chen T., Vallese F., Kapoor K., Clarke O.B., Tajkhorshid E. (2024). Atomic-resolved description of anion binding and the alternating access mechanism of anion exchanger 1 (band 3). Biophys. J..

[119] Lv H., Cao Y., Zhu J., Liang Q. (2024). Molecular insights into the effect of cholesterol on the binding of bicarbonate ions in band 3 protein. Langmuir.

[120] Hansen S.B., Tao X., MacKinnon R. (2011). Structural basis of PIP2 activation of the classical inward rectifier K^+^ channel Kir2.2. Nature.

[121] Pyle E., Guo C., Hofmann T., Schmidt C., Ribiero O., Politis A. (2019). Protein-lipid interactions stabilize the oligomeric state of BOR1p from Saccharomyces cerevisiae. Anal. Chem..

[122] Rivera-Santiago R., Harper S.L., Sriswasdi S., Hembach P., Speicher D.W. (2017). Full-length anion exchanger 1 structure and interactions with Ankyrin-1 determined by zero length crosslinking of erythrocyte membranes. Structure.

[123] Jennings M.L. (2021). Cell physiology and molecular mechanism of anion transport by erythrocyte band 3/AE1. Am. J. Physiol. Cell Physiol..

[124] Kollert-Jons A., Wagner S., Hubner S., Appelhans H., Drenckhahn D. (1993). Anion exchanger 1 in human kidney and oncocytoma differs from erythroid AE1 in its NH2 terminus. Am. J. Physiol..

[125] Ideguchi H., Okubo K., Ishikawa A., Futata Y., Hamasaki N. (1992). Band 3-Memphis is associated with a lower transport rate of phosphoenolpyruvate. Br. J. Haematol..

[126] Yannoukakos D., Vasseur C., Driancourt C., Blouquit Y., Delaunay J., Wajcman H. (1991). Human erythrocyte band 3 polymorphism (band 3 Memphis): characterization of the structural modification (Lys 56—Glu) by protein chemistry methods. Blood.

